# Cellular zinc metabolism and zinc signaling: from biological functions to diseases and therapeutic targets

**DOI:** 10.1038/s41392-023-01679-y

**Published:** 2024-01-03

**Authors:** Bonan Chen, Peiyao Yu, Wai Nok Chan, Fuda Xie, Yigan Zhang, Li Liang, Kam Tong Leung, Kwok Wai Lo, Jun Yu, Gary M. K. Tse, Wei Kang, Ka Fai To

**Affiliations:** 1 Department of Anatomical and Cellular Pathology, State Key Laboratory of Translational Oncology, Prince of Wales Hospital, The Chinese University of Hong Kong, Hong Kong, China; 2 State Key Laboratory of Digestive Disease, Institute of Digestive Disease, The Chinese University of Hong Kong, Hong Kong, China; 3 CUHK-Shenzhen Research Institute, The Chinese University of Hong Kong, Shenzhen, China; 4 Department of Pathology, Nanfang Hospital and Basic Medical College, Southern Medical University, Guangdong Province Key Laboratory of Molecular Tumor Pathology, Guangzhou, China; 5 Institute of Biomedical Research, Taihe Hospital, Hubei University of Medicine, Shiyan, China; 6 Department of Pediatrics, The Chinese University of Hong Kong, Hong Kong, China; 7 Department of Medicine and Therapeutics, The Chinese University of Hong Kong, Hong Kong, China

**Keywords:** Tumour angiogenesis, Cancer therapy, Cell biology

## Abstract

Zinc metabolism at the cellular level is critical for many biological processes in the body. A key observation is the disruption of cellular homeostasis, often coinciding with disease progression. As an essential factor in maintaining cellular equilibrium, cellular zinc has been increasingly spotlighted in the context of disease development. Extensive research suggests zinc’s involvement in promoting malignancy and invasion in cancer cells, despite its low tissue concentration. This has led to a growing body of literature investigating zinc’s cellular metabolism, particularly the functions of zinc transporters and storage mechanisms during cancer progression. Zinc transportation is under the control of two major transporter families: *SLC30* (ZnT) for the excretion of zinc and *SLC39* (ZIP) for the zinc intake. Additionally, the storage of this essential element is predominantly mediated by metallothioneins (MTs). This review consolidates knowledge on the critical functions of cellular zinc signaling and underscores potential molecular pathways linking zinc metabolism to disease progression, with a special focus on cancer. We also compile a summary of clinical trials involving zinc ions. Given the main localization of zinc transporters at the cell membrane, the potential for targeted therapies, including small molecules and monoclonal antibodies, offers promising avenues for future exploration.

## Introduction

As an crucial trace element, zinc is critical for numerous biological functions, and its imbalance has been linked to a variety of pathologies, including cancer.^1,2^ Understanding the intricacies of zinc metabolism at the cellular level, including encompassing the absorption, intracellular trafficking, utilization, storage, and expulsion of zinc, can shed light on the various effects of zinc in cell physiology and pathology.^3^ Zinc, an essential component in the regulation of cellular homeostasis, is receiving increasing attention for its role in cancer.^4,5^

Significantly, an extensive body underscores the crucial role of zinc homeostasis across various biological systems. Zinc is estimated to bind to around 3000 proteins in vivo, representing about 10% of the human proteome,^6^ with over 3% of genes in human bodies encoding proteins containing zinc finger domains. Consequently, zinc assumes a pivotal position during numerous physiological processes, including cell cycle progression,^7–9^ immune functions,^10^ meiosis,^11^ and many other physiological procedures. Intracellular zinc metabolism and zinc signaling are exceptionally precise. Cytoplasmic free zinc concentration remains within the picomolar range, while the overall zinc level is estimated to be about 200–300 μM.^12^

Cellular zinc homeostasis is delicately regulated by a network of proteins, which includes the solute carrier (SLC) families *SLC30* (ZnT) and *SLC39* (Zrt- and Irt-like proteins/ZIP), as well as the zinc-binding (MTs).^2,13^ These proteins are crucial in the maintenance of cellular zinc homeostasis. Traditionally, two transporter family members operate opposite directions to achieve this equilibrium. The *SLC30* family, encoding ZnT proteins, facilitates zinc efflux through translocating zinc from the cytoplasm to the lumen of organelles or the extracellular space.^1^ Conversely, the *SLC39* family, also known as the ZIP family, functions in zinc influx, transporting zinc into the cytoplasm from the extracellular space of the cell or the intracellular storage compartment, effectively elevating zinc levels.^14^ Meanwhile, MTs majorly handle zinc storage within the cell, safeguarding against potential toxicity while ensuring availability when required.^13^ Increasingly, cellular zinc metabolism has been linked to disease progression. This review will explore the potential role of cellular zinc metabolism in biology, tumorigenesis, and drug applications.

## Regulation of cellular zinc signaling

### Zinc distribution

Zinc is prevalent in various human tissues. Adults typically possess a zinc content ranging from 1.4 to 2.3 g.^15^ Approximately 85% of zinc resides in the muscles as well as the bones. Besides, about 11% of zinc is in the skin and liver. The remaining 4% of zinc was scattered in other tissues.^16^ Notably, the maximum zinc concentration has been found in the retina and choroid of the eye.^17^ Additionally, zinc is found in considerable amounts in the prostate, bones, liver, and kidneys.^18^

Notably, most of the zinc is intracellular. Approximately 30–40% of the content resides in nuclei of cells, with approximately half distributed across the cytosol, organelles, and specific vesicles, while the remaining zinc is associated with cell membranes.^19^ Based on current research, the total pool of zinc, encompassing both intracellular and extracellular compartments, can be distinguished into three distinct categories.^20,21^ Firstly, the term “Immobile zinc” refers to zinc that is firmly bound to metalloproteins or metalloenzymes, serving as either a structural component or a cofactor. This form of zinc is stable and non-reactive. Secondly, “Mobile reactive zinc” or “labile zinc” is loosely associated with low molecular weight ligands and MTs. This form is exchangeable and reactive. Notably, this mobile form constitutes about 5% of all intracellular zinc, playing a pivotal role in zinc transfer reactions and signaling processes.^22,23^ Lastly, the “free zinc” pool is another reactive form of the element. In mammalian cells and in extracellular fluids, however, the concentration of this zinc is quite low, with values oscillating between roughly 5 pM and 1 nM.^24^

MTs, colloquially referred to as “zinc storage,” maintain intracellular free zinc levels through their interaction with cysteine.^25,26^ In addition to MTs, members of the zinc transporter family, including ZIPs and ZnTs, play a critical role in managing zinc homeostasis. Remarkably, the cellular zinc transport activity of ZnT7 is crucial for regulating the localization of ERp44 within the Golgi apparatus, a specific subcellular organelle.^27^ Notably, many secretory enzymes obtain essential cellular zinc in the Golgi complex. Moreover, as a molecular chaperone acting in the early secretory pathway, ERp44 can bind to zinc to control the protein binding and release, thereby managing protein transport and stability.

In recent times, the essential and multifaceted function of zinc as a signaling molecular has attracted significant attention. The generation of zinc signals arises from three main sources: vesicular exocytosis, zinc transport facilitated by zinc transporters for entry or exit from the cell or organelle, and the binding or dissociation of MTs with zinc. These aspects will be expounded upon in the subsequent sections.

### Intracellular zinc signaling

The total concentrations of zinc in cells range from 200–300 μM,^12^ whereas the eukaryotic labile (“free”) zinc concentration is in the picomolar range, as mentioned earlier for each specific cell type.^24^ Notably, the cytoplasm contains minimal free zinc since intracellular zinc is mainly sequestered in organelles like the ER, Golgi apparatus, and mitochondria, the so-called zinc store.^28^ Growing evidence suggests that zinc functions not only as a neurotransmitter for cell-to-cell communication but also as an intracellular signaling molecule, facilitating the transduction of various signaling cascades in response to extracellular stimuli. This has led to the concept of zinc as the “calcium of the 21^st^ century”.^29^

As previously mentioned, there are two pathways for intracellular zinc ion release, namely from intracellular zinc stores or zinc/sulfate sites in proteins, such as in MTs. Transient zinc increases may arise from various mechanisms, including efflux from vesicles known as zincosomes,^30^ or changes in cellular redox potential facilitated by cytosolic proteins.^31^ It is important to know that, in most cases, zinc signaling arises from the disturbance of intracellular zinc homeostasis, transiently and rapidly. The functioning of zinc ion transporters and MTs in the cell plays a role in maintaining cytoplasmic zinc homeostasis, which is referred to as “buffering” and “muffling”, two essential parameters that determine the availability and signaling processes of zinc.^32^ Specifically, “buffering” involves zinc binding by proteins like MT, which helps maintain zinc concentration at the pM range in the cytosol.^33^ The biology of MTs is characterized by zinc binding, movement within the cell, and transportation of zinc to various cellular compartments, including extracellular, endosomal, nuclear, and mitochondria.^34^ The chelating agent will accelerate this process, but if gene expression such as MT is involved, “buffering” would be slow.^35^ “Muffling”, on the other hand, is responsible for modulating transient changes in zinc concentrations under unsteady state conditions of cells, eventually restoring the cytosolic concentrations to their resting levels.^12,36^ In the “muffling” process, zinc transporters regulate cellular zinc by importing, distributing, exporting, and providing zinc for zinc-dependent proteins.^36^ For example, ZnT5,6 loads zinc for the enzymes of the secretory pathway,^37,38^ while ZnT2,3,8 provides zinc for the exocytotic vesicles.^39–41^ Moreover, MT is also responsible for zinc muffling by moving and sequestering zinc to cellular compartments, thus controlling kinetically ion concentrations.^42^

**Fig. 1:**
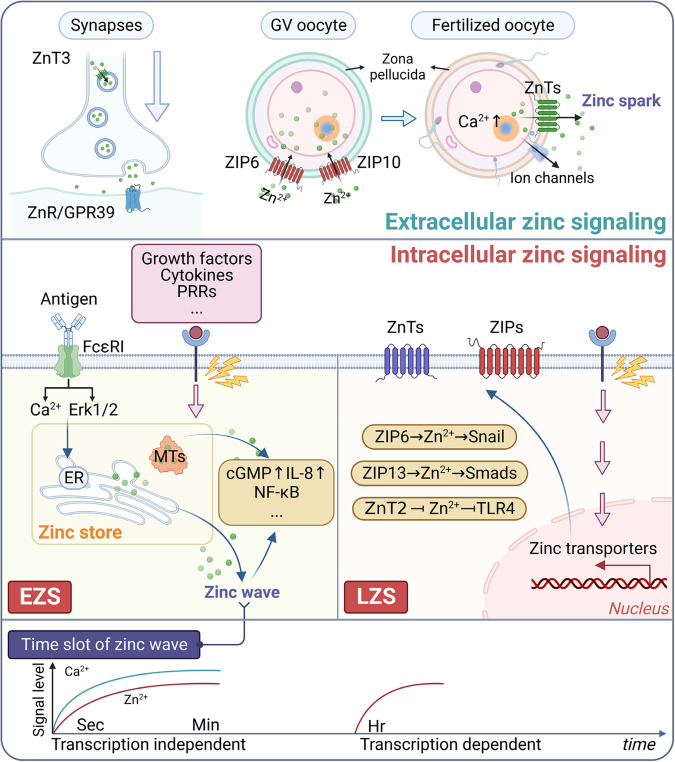
Zinc signaling in the intracellular and extracellular regions. Zinc extracellular signaling is mainly involved in the physiological functions of neurosynapses and germ cells. In contrast, intracellular zinc signaling is primarily divided into two parts, EZS and LZS, which exert biological functions by activating downstream pathways, such as inflammatory signaling. Interestingly, the endoplasmic reticulum releases zinc to generate a specific zinc wave, observed within several minutes after FcεRI stimulation in mast cells. EZS early zinc signaling, LZS late zinc signaling. Green dots represent zinc

In terms of time series, intracellular zinc serves as a second messenger, and its concentration transients are divided into two main types: early (fast) zinc signaling (EZS) and late zinc signaling (LZS)^43^ (Fig. 1). The study further confirmed that EZS is transcription-independent, occurring over a timescale ranging from seconds to minutes, known as the “zinc wave”.^36^ This phenomenon was first observed in mast cells and results from Fcε epsilon receptor I (FcεRI) stimulation, causing a transient, transcription-independent increase in intracellular zinc.^44^ The “zinc wave” originates in the perinuclear region, including the ER, and depends on calcium influx and MEK activation. However, the precise mechanism of the “zinc wave” in cells remains poorly understood. In contrast, LZS requires the transcription of zinc transport proteins and has longer-lasting effects lasting for hours. In this case, diverse extracellular stimuli, including cytokines and growth factors, indulge the transcriptional modulation of zinc-associated proteins like ZIPs and ZnTs. Consequently, intracellular zinc homeostasis alterations regulate downstream molecular objectives, in addition to protein kinase C (PKC), ERK1/2 activation leading to neuronal cell death, cAMP-dependent protein kinase (PKA), Ca/calmodulin-dependent protein kinase II (CaMKII), phosphodiesterases (PDEs), protein tyrosine phosphatases (PTPs), and transcription factors, such as NF-κB.

Notably, the elevated intracellular zinc has a bidirectional effect. On the one hand, zinc participates in various cellular signaling pathways, contributing to processes such as cell proliferation and differentiation.^45–48^ For example, zinc promotes embryonic central nervous system (CNS) development by affecting STAT1 and STAT3 signaling pathways.^49^ Interestingly, it has been shown that zinc has a more significant role in hematopoiesis than iron, at least in early hematopoietic stem cells.^50^ In immune function-related signaling, zinc enhances the development of regulatory T cells, as induced by the transcription factor Foxp3.^51,52^ On the other hand, excessive intracellular zinc accumulation can lead to apoptosis. Mitochondrial-derived zinc accumulation can impair mitochondrial structure and function, negatively impacting animal development and longevity in *Caenorhabditis elegans*.^53^ Studies have also demonstrated that intracellular zinc release might occur as a response to oxidative or nitrosative stress, which could lead to the release of zinc from MT, a zinc buffer protein, thereby promoting apoptotic processes.^54,55^ Furthermore, In a specific cell death pathway, the release of zinc and calcium within neurons leads to the subsequent phosphorylation of the potassium channel Kv2.1.^56,57^ In conclusion, despite low intracellular free zinc concentrations, intracellular zinc signaling plays a broad and vital role in physiological functions.

### Extracellular zinc signaling

Extracellular zinc is a significant signaling mediator in endocrine, paracrine, and autocrine systems.^58,59^ It serves as a ligand for various receptor channels on the plasma membrane, including the zinc sensing receptor (ZnR/GPR39) that regulates neuronal excitation,^60^ N-methyl-D-aspartate (NMDA) receptors,^61^ α-amino-3-hydroxy-5-methyl-4-isoxazolepropionic acid (AMPA) receptors,^62^ voltage-dependent calcium channels (VDCC),^63^ and γ-aminobutyric acid A (GABAA) receptors.^64,65^ The progress within cell biology and chemistry has emphasized the presence and function of free or labile zinc in cellular responses, especially its neurotransmitter role in synaptic vesicles.^29,66,67^ Fluctuations within brain zinc concentrations, corresponding to physiological experiences and long-term memories, indicate that free zinc is strongly associated with neurotransmitter performance.^68^ Moreover, zinc released from the synapse directly activates a G-protein coupled receptor (mZnR/GPR39), sensing changes in extracellular zinc concentration and consequently regulating neuronal excitation.^69^

In addition, fertilized mammalian embryos release zinc sparks.^8,11^ The exocytotically released zinc ions coordinate with cellular calcium transients, modifying the structure of the zona pellucida to prevent polyspermy (Fig. 1).

### Zinc signaling and tumorigenesis

Under normal circumstances, zinc concentration meets the demands of bioenergetic, synthetic, and catabolic, essential for manifesting the cells’ current activities, e.g., function, growth, and proliferation. Several mechanisms explain the antitumor function of zinc, encompassing DNA damage, DNA repair, immune function, oxidative stress, and inflammation.^70–72^ As cell activity changes, its metabolism must be adjusted to accommodate any newly established biological energy/synthetic/catabolic requirements. Changes in zinc concentrations beyond the cell’s ability to coordinate can lead to tumorigenesis, as zinc provides the bioenergetic/ synthetic requirements of malignancy, such as the aberrant expression of zinc transporters and dysregulation of MTs binding proteins.^73–75^

Indeed, zinc activation of two mitogen-activated protein kinase (MAPK) pathways linked to tumorigenesis, namely extracellular signal-related kinase (ERK) and c-Jun N-terminal kinase (JNK),^44^ plays a significant role. These MAPKs, including ERK and JNK, are serine/threonine protein kinases that regulate cell proliferation, differentiation, and apoptosis in tumorigenesis.^76^ Regarding the late zinc signaling, STAT3 stimulates the transcriptional activity of ZIP6 in zebrafish.^70^ As a result, STAT3-dependent ZIP6 expression leads to downstream activation of the transcriptional repressor Snail, which contributes to the epithelial-mesenchymal transition (EMT) during embryonic development and is associated with tumor metastasis mechanisms (Fig. 1). Similarly, ZIP4 induces EMT-promoting migration and invasion through the PI3K/Akt signaling pathway in nasopharyngeal carcinoma (NPC).^77^ Additionally, elevated expression of ZIP13 activates the Src/FAK pathway, leading to increased expression of pro-tumor metastatic genes but decreased expression of tumor suppressor genes in ovarian cancer.^73^ Overall, cancer cells appear to require stimulation of oncogenic pathways by zinc to maintain their aggressiveness.

Obviously, cellular zinc signaling benefits from the storage and release of organelles and subcellular structures, which are precisely regulated by the zinc transporters and MTs. Thus, maintaining zinc homeostasis requires a complex intracellular collaboration of these functional proteins. Hypothetically, would normal cells transform into cancer if zinc homeostasis were disrupted? A plethora of studies have substantiated that dysregulation of zinc transporter proteins not only affects cell proliferation and apoptosis but also induces alterations in various signaling pathways, thus promoting cancer progression.^73–75^ Remarkably, the lysosomal cation channel MCOLN1 has been identified as a crucial mediator of zinc influx into the cytoplasm, thereby finely controlling oncogenic autophagy in cancerous cells.^78^ Additionally, alterations in zinc homeostasis have been shown to modulate the tumor immune microenvironment, exerting a significant influence on cancer progression.^79^ Furthermore, the involvement of zinc in heavy metal detoxification implies that its disruption could adversely affect detoxification pathways, thereby leading to cellular stress and subsequent cancer development.^26^ In conclusion, the intricate link between zinc homeostasis and cancer is an emergent field that warrants further exploration to fully elucidate the underlying mechanisms that govern the transition from disrupted zinc homeostasis to tumorigenesis.

## Regulation of cellular zinc metabolism

### The basic knowledge of zinc transporters

#### ZIPs

The *SLC39* family comprises four distinct groups based on amino acid sequence similarities: subfamily I (ZIP9); subfamily II (ZIP1, 2, and 3); the LIV-1 subfamily (ZIP4, 5, 6, 7, 8, 10, 12, 13, and 14); and the gufA subfamily containing ZIP11.^80^ All ZIP proteins have eight transmembrane (TM) domains with conserved histidine residues within TM 4 and 5, believed to be involved in zinc transportation. The C-terminal and N-terminal ends of ZIP are located either on the cell surface or within the lumen of the organelle.^81,82^ Members of the LIV-I family, with the exception of ZIP13, are anticipated to possess one significant, extracellular N-terminal domain, suggested to function as extracellular zinc sensors. Recently, research has provided insights into the detailed structure of ZIP transporters, including a high-resolution 3.05 Å cryo-electron microscopy structure of a ZIP-family transporter from Bordetella bronchiseptica acquired in an inward-facing, inhibited conformation.^83^ Each protomer of this homodimeric transporter comprises nine transmembrane helices and three metal ions. In this architecture, two metal ions create a binuclear pore structure, and the third ion is located at an egress site facing the cytoplasm. Notably, this egress site is covered by a loop, with two histidine residues on this loop interacting with the egress-site ion, crucially regulating its release. Understanding the structure and function of ZIP transporters may offer valuable insights for developing new therapeutic strategies targeting zinc transporters to treat various human diseases.

The ZIPs are typically synthesized on ribosomes attached to the endoplasmic reticulum (ER) and later transported to various intracellular compartments.^84^ Similar to other protein expressions, unstable ZIP mutant proteins are often identified in the ER. Subsequently, they undergo retro translocation and degradation by cytosolic proteasomes in a ubiquitin-independent manner, as seen in the case of ZIP13 mutant.^85^ Apart from the intracellular localization of certain ZIP members, the majority of ZIP transporters are positioned on the plasma membrane, facilitating metal ion uptake into cells. ZIP7 is situated in the Golgi apparatus and ER, while ZIP13, evolutionarily closest to ZIP7, is localized in the Golgi apparatus and cytoplasmic vesicles.^85,86^ ZIP13 is responsible for mobilizing zinc from the lumen of these compartments and plays crucial roles in cellular signaling, including the BMP/TGF-β signaling pathway, by regulating the nuclear translocation of Smad proteins and maintaining ER homeostasis.

The expression levels of numerous ZIPs, such as ZIP1, 3, 4, 8, and 12, at the cell surface, are modulated by the available concentrations of zinc.^80^ ZIP10 serves as a cell surface zinc importer.^87^ The transcription of ZIP10 is upregulated in zinc-depleted cells^88^ and downregulated in zinc-excess conditions. The regulation of zinc transcription is mediated by pausing Pol II transcription through the action of metal response element-binding transcription factor-1 (MTF-1). Furthermore, the positioning of certain ZIP proteins varies with zinc supply and specific physiological states. During adequate zinc intake, Zip5 aligns at the basolateral plasma membrane in polarized cells.^89^ In a parallel manner, ZIP14 moves to the mouse hepatocyte’s sinusoidal membrane during sharp inflammatory events.^90^ As a result, this boosts zinc absorption as part of the immediate response to inflammation.

While ZIP members are known for their primary role in transporting zinc, they can also mobilize other metals such as manganese and cadmium.^91–94^ Biochemical studies have shown that ZIP8, in particular, can transport cadmium and manganese.^95–97^ The expression of ZIP8 mRNA is upregulated by cadmium in an NF-κB-dependent manner, contributing to the risk of cadmium-mediated lung toxicity exposed to cigarette smoke.^98^ ZIP14 is evolutionarily closely related to ZIP8.^99^ Similar to ZIP8, ZIP14 has the ability to mobilize various divalent cations, including cadmium and manganese.^100^ Moreover, ZIP14 and ZIP8 are capable of transporting iron.^101,102^ ZIP14 plays a crucial role as an iron transporter in vivo, especially under iron overload conditions.^103^ ZIP14’s capability to transport non-transferrin-bound iron (NTBI) is considered a vital contribution to iron homeostasis.^100^ Interestingly, ZIP14 possesses two spliced variants: ZIP14A and ZIP14B. These variants are present on the plasma membrane and are involved in zinc uptake. In polarized cells, ZIP14A and ZIP14B are exclusively located on the apical surface.^99^

#### ZnTs

**Fig. 2:**
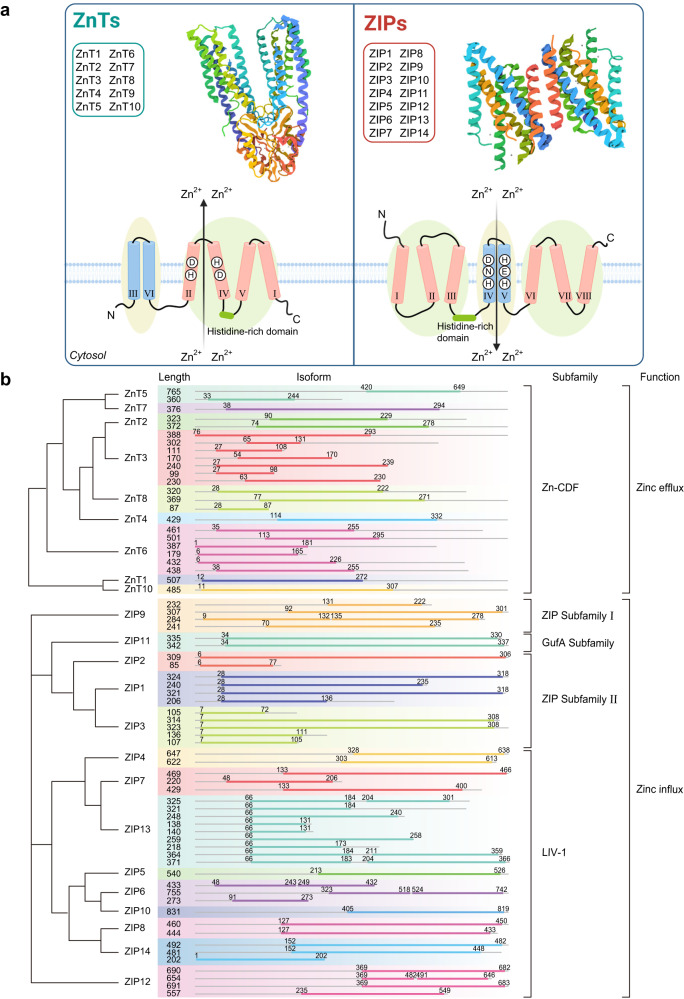
The protein structure and gene family evolution of zinc transporters. **a** Cartoon of predicted structures of ZnT and ZIP transporter proteins. The picture on the left shows an atomic model of ZnT, which is the helical reconstruction of YiiP based on X-ray structure (PDB ID code: 7y5g). In detail, the schematic topology of ZnT transporters is proposed based on the three-dimensional structure of Escherichia coli homolog YiiP. ZnTs most likely have six TM domains divided into two bundles. Specifically, one of the ZnT’s bundles contains four TM domains (MI, MII, MIV, and MV), and the other one comprises two TM domains (MIII and MVI). Each of the former bundle’s domains can independently bind zinc, tetrahedrally coordinated by two D (aspartate) and two H (histidine) in the mammalian homologs. Similarly, the figure on the right presents putative TM domains of the ZIP family (PDB ID code: 7z6m). Moreover, the topology structure of ZIP is displayed, composed of 8 TM domains with a large N-terminal domain and a small C-terminal. The spatial distribution shows that it consists of three parts, the left and right parts each contain three TM domains (red), and in the middle are two TM domains (blue). Zinc could bind to the active site of TM domain IV and V, containing conserved HND (histidine, asparagine, aspartate) and HEH (two histidines and one glutamic acid) motifs, respectively. **b** Gene family evolutionary tree and isoform of the ZnTs and ZIPs. The lengths of the different isoforms are labeled in the front of the isoforms, and the color lines indicate the functional domain locations of each isoform. ZnTs belong to the Zn-cation diffusion facilitator (CDF) family, responsible for transporting zinc from intracellular to extracellular. ZIPs are divided into four subfamilies, namely ZIP subfamily I (ZIP9), GufA subfamily (ZIP11), ZIP subfamily II (ZIP1-3), and LIV-1 subfamily (ZIP4-8, ZIP10, ZIP12-14)

The ZnT family belongs to the cation diffusion facilitator (CDF) family of proteins. Most ZnTs are located within organellar membranes, serving various functions, such as filling vesicular zinc stores, supplying organelles with zinc, and loading exocytotic vesicles with zinc for essential biological processes. The structure of ZnT proteins is inferred from the *Escherichia coli* homologs of YiiP,^104^ which have six TM helices (TM helices I-VI) and their N- and C-termini situated on the cytoplasmic side.^104,105^ ZnT5, on the other hand, possesses an unusually long N-terminal region with nine putative TM domains.^106^ ZnT transporters are also expected to contain a conserved zinc-binding site on TM helices II and V, with critical residues determining their metal specificity.^14,105^ Remarkably, ZnT10 demonstrates the molecular features of a manganese transporter, likely attributed to its possession of an Asn residue rather than His in the TM helix II.^104^ Furthermore, the length and amino acid sequence of the initial TM structural domain of ZnT proteins, known for containing subcellular targeting signals, display substantial variations among different ZnT proteins. Based on their protein sequence similarities, the ZnT family members can be categorized into four groups: (1) ZnT6 and ZnT9, (2) ZnT1 and ZnT10, (3) ZnT2-4 and ZnT8, and (4) ZnT5 and ZnT7. Intriguingly, members belonging to the same subfamily exhibit similar cellular locations and functional characteristics^1^ (Fig. 2).

Functionally, in the *SLC30* family, ZnT1 functions primarily as a zinc exporter on the cell membrane, transporting cytoplasmic zinc ions across the membrane to the extracellular space, while other ZnT proteins are situated on the membranes of intracellular organelles.^107^ Besides, ZIP10 and ZnT1 are involved in renal zinc reabsorption.^108,109^ Members of the subfamily II of the SLC30 proteins (ZnT2, ZnT3, ZnT4, and ZnT8) play a major role in secretory tissues, with ZnT3 involved in neurotransmission, ZnT8 in insulin storage, ZnT4 in prostate secretion, and ZnT2 in lactation.^40,108,110,111^ Besides, Additionally, TMEM163 is a recently discovered zinc transporter with a predicted transmembrane domain structure and function similar to the CDF protein superfamily.^112^ Some posit that TMEM163 could be a novel member of the mammalian ZnT transporter proteins.^113^ Recent discoveries indicate its significant role in maintaining zinc balance in both nerves and blood.^114–116^

### The basic knowledge of MTs

Mammalian MTs are a superfamily of nonenzymatic polypeptides that typically consist of 61–68 amino acids.^25^ They are characterized by a high cysteine content, accounting for approximately 30% of their amino acids, while aromatic amino acids are absent, and histidine residues are sparsely present. However, they contain abundant thiol groups that enable them to bind to heavy metals. MTs, with their abundant thiol groups, have the capacity to bind up to 7 zinc atoms: 3 zinc atoms in the β domain and 4 zinc atoms in the α domain.^117,118^ This unique capability enables MTs to function as a cellular zinc reserve. It is crucial to highlight that while MTs can bind other essential metals such as copper and nonessential metals like cadmium, the predominant form in human tissue is zinc-bound MT.

Human MTs can be classified into four classes, namely MT1 to MT4, comprising a total of eleven functional isoforms, with eight of them belonging to class 1.^3^ MT1 and MT2 are the predominant isoforms distributed throughout the human body and expressed in various organs. Conversely, MT3 is predominantly present in the CNS, while MT4 is primarily found in the skin and other stratified epithelium, representing the minor isoforms.^119^ All isoforms have an approximate molecular weight of 7 kDa and lack aromatic amino acids. Moreover, they consist of twenty cysteine residues, endowing MTs with distinctive characteristics due to the properties of thiol groups.^120^ Additionally, the transcription of MT1/2 genes is governed by MTF-1, a zinc finger transcription factor that regulates the expression of metal-responsive genes. Zinc is notably the sole known metal to activate MTF-1; however, studies propose that oxidative stress might also contribute to MTF-1 activation.^121^ MTF-1 is involved in regulating the zinc-responsive transcription of ZnT1 and ZnT2 and inhibiting the expression of ZIP10,^87,122,123^ emphasizing its vital role in zinc homeostasis.

**Fig. 3:**
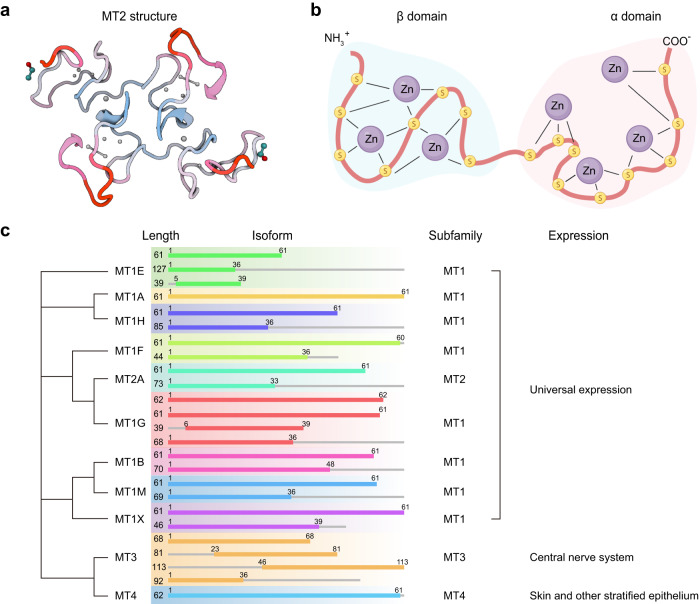
The protein structure and gene family evolution of MTs. **a** Diagram of the predicted structure of the MT2 protein, which is modeled from the reconstructed X-ray structure (PDB ID code: 4mt2). The crystallographic structure of rat liver metallothionein has been accurately determined at a resolution of 2.0 Å, achieving a low R-value of 0.176 for all observed data. **b** Schematic representation of zinc binding in MTs. MTs contain abundant thiol groups capable of binding with heavy metals. Due to the high thiol content, MTs can bind up to 7 zinc atoms, with 3 zinc atoms located in the β domain and 4 zinc atoms in the α domain. **c** Gene family evolutionary tree and isoforms of MTs are depicted in Figure X. The isoform lengths are labeled in front of each isoform, and color lines indicate the position of the functional domain of each isoform. MTs are categorized into four subfamilies: MT1 (including *MT1A*, *MT1B*, *MT1E*, *MT1F*, *MT1G*, *MT1H*, *MT1M*, and *MT1X*), MT2 (including *MT2A*), MT3, and MT4. While MT1 and MT2 are universally expressed, MT3 is primarily expressed in the central nervous system, and MT4 is predominantly expressed in the skin and other stratified epithelium tissues

In humans, MTs are structurally encoded by a family of genes located on chromosome 16q13, comprising at least 11 functional members: the *MT1* genes consist of 18 isoforms, including 10 functional genes (*MT1A, MT1B, MT1E, MT1F, MT1G, MT1H, MT1M, and MT1X*) and 8 pseudogenes (*MT1CP, MT1DP, MT1JP, MT1L, MT1LP, MT1XP1, MT1P3, and MT1P1*), in addition to *MT2* (also known as MT2A), *MT3*, and *MT4*^55,119^ (Fig. 3). Remarkably, as the zinc store, MT can act as both zinc receptor and zinc donor, like two sides of the same coin.^118,124^

## Role of cellular zinc metabolism under physiological conditions

### The physiological role of zinc transporters

#### Supporting immune function

T cells are a critical component of the immune system.^125^ Among the 14 ZIP family members, ZIP6, 8, and 13 are highly expressed in human CD4^+^ T cells, with ZIP6 predominantly localized to lipid rafts involved in the immune synapse (IS) formation following T cell receptor (TCR) stimulation.^126^ Notably, the tyrosine phosphorylation of ZIP6 was observed to increase after five minutes of TCR stimulation due to its interaction with Zap70, a crucial kinase involved in early TCR signaling. In addition, the transcriptional activity of ZIP6 leads to zinc influx, promoting the expression of MTs, which plays a crucial role in supporting T cell proliferation and is essential for T cell survival and expansion in the elderly.^127,128^ ZIP8 and ZIP13 are primarily expressed on the lysosome and ER/Golgi membrane of T cells, respectively.^86,129^ During T cell activation, ZIP8 facilitates zinc transport from the lysosome to the cytoplasm, resulting in increased production of IFN-γ. Notably, ZIP8 expression can be induced in response to lipopolysaccharide (LPS) stimulation,^130,131^ leading to enhanced IL-1β production downstream of the mTORC1S6K pathway.^132^ Moreover, ZIP8 is a downstream target gene of NF-κB, which negatively regulates pro-inflammatory responses through zinc-mediated downregulation of Iκκ activity.^130^ Comparatively, the deficiency of ZIP8 has a substantial impact on zinc influx in effector T cells and results in reduced TCR-mediated signaling, including NF-κB and MAPK signaling, which are involved in the differentiation of T helper (Th)17 cells.^133^ Similarly, mice lacking ZIP3 exhibit decreased CD4^+^ CD8^+^ double-positive (DP) thymocytes but increased CD4^+^ and CD8^+^ single-positive thymocytes, indicating its role in regulating T cell development.^134^ These findings open up new possibilities for immunotherapy to improve the prognosis by modulating the zinc transporter family genes on tumors or immune cells.

**Fig. 4:**
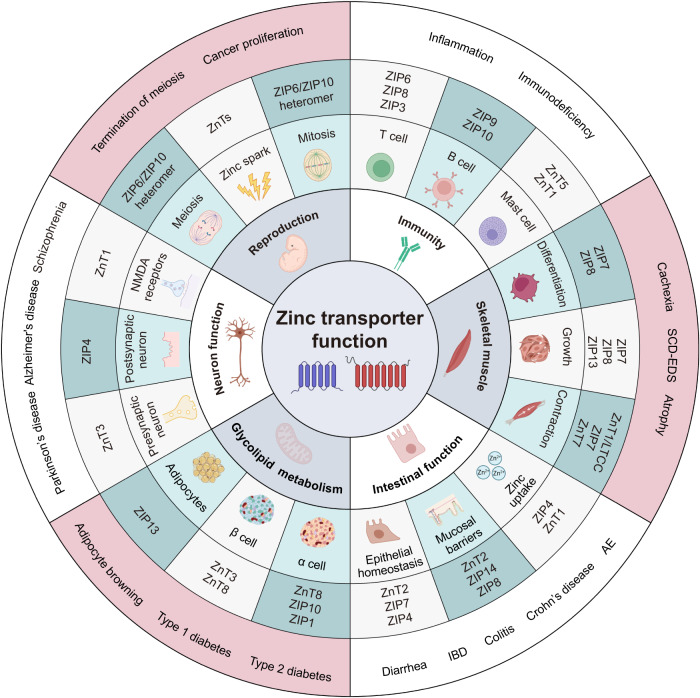
The main physiological functions of zinc transporters. The zinc transporter functions are basically classified into six parts: immunity, reproduction, muscle, intestinal function, glycolipid metabolism, and neuron function. Further, the function represented by each sector is mainly divided into three parts. Each part corresponds to specific zinc transporters. The outermost circle represents diseases and cancers caused by malfunctioning zinc transporters. SCD-EDS spondylocheirodysplastic ehlers-danlos syndrome, AE acrodermatitis enteropathica, IBD inflammatory bowel disease

Undoubtedly, the adaptive branch of the immune system relies on both B cells and T cells.^135^ ZIP9 and ZIP10 play essential roles in B cell receptor signaling pathways, influencing B cell activation^136,137^ (Fig. 4). The release of zinc in B cells originates from the Golgi apparatus, with ZIP9 playing a crucial role as the zinc transport participant.^136^ ZIP10, on the other hand, plays different roles in the early and late stages of B cell development, regulating distinct signaling cascades. The expression of ZIP10 is mechanistically regulated in a STAT3/STAT5-dependent manner, promoting early B cell survival by inhibiting caspase activation.^137^ Additionally, ZIP10 deficiency in mature B cells has been shown to attenuate both T cell-dependent and -independent immune responses in vivo.^138^ ZIP10 functions as a positive regulator of CD45R in B cell antigen receptor signaling transduction, playing a crucial role in setting a threshold for human immune responses. In hepatocellular carcinoma (HCC) cell lines, ZIP10 expression was found to be positively correlated with tumor-infiltrating lymphocytes and certain immune checkpoints, including CTLA4, TIM3, and TGFB1.^139^ Moreover, ZIP10 is essential for zinc homeostasis within macrophages, where zinc is involved in antimicrobial responses.^140^ activated macrophages, while crucial for immune responses, can also release large quantities of inflammatory cytokines, which may have the potential to harm the host.^141^ ZIP10 was identified as a significant zinc importer in macrophages that activates macrophages and promotes cytokine expression.^142^ Zinc deficiency (ZD) caused by knocking down ZIP10 leads to cytoplasmic p53 accumulation and nuclear translocation of AIF, ultimately triggering apoptosis.^142^ Thus, targeting ZIP10 could be a promising approach to protect the liver from inflammation damage.

Notably, sepsis is an acute systemic infection triggered by the invasion of pathogenic bacteria into the blood circulation and the production of toxins.^143^ Circulating zinc levels lower than expected have been linked to high mortality in sepsis patients, with MT and ZIP8 identified as two of the most highly upregulated genes in non-survivors.^144^ ZIP8, in particular, has been found to be the most significantly upregulated transporter in response to cytokines, bacteria, and sepsis, indicating its unique role in innate immune function.^130,145,146^ As the closest homolog of ZIP8, ZIP14 also participates in response to sepsis and is implicated in the beneficial anti-inflammatory effects of supplemental dietary zinc during sepsis, indicating its potential as a therapeutic target.^147^ Additionally, the existence of “zinc waves” in mast cells provides further evidence of the involvement of zinc transporters in immune functions.^44^ The release of zinc from the ER is likely mediated by ZIP7, as ZIP7 predominantly resides in the ER, and silencing ZIP using siRNA prevented the occurrence of the zinc wave.^148,149^ Besides, ZnT1/L-type voltage-gated calcium channels (LTCCs) also contribute to the zinc wave, which interacts with ZnT1 and modulates the zinc influx from extracellular space into the cytoplasm.^150–152^

#### Assistance of reproduction

During meiotic maturation, total intracellular zinc increased by ~50%. After fertilization, zinc-rich oocytes induced zinc sparks, which decreased zinc concentration by approximately 20%. The role of zinc sparks requires further investigation, but some evidence suggests that these changes in zinc levels are crucial for subsequent developmental steps and may play a role in zinc-dependent processes regulating oocyte exit from meiosis I.^11,153^ The ZIP transporter family is believed to regulate zinc influx, and ZIP6 and ZIP10, which share 43.5% sequence identity and are on the same clade of the ZIP family phylogenetic tree,^154^ are highly expressed in the oocyte during the window of meiotic maturation^155^ (Fig. 4). The ZIP6/ZIP10 heteromer is also critical for triggering zinc-mediated mitosis,^156^ forming a zinc-dependent mitotic complex consisting of ZIP6, ZIP10, pS^727^STAT3, and pS^38^Stathmin, which play roles in proven mitotic pathways. As an illustration, they are involved in processes like stathmin- reliant microtubule reorganization or HistoneH3-mediated chromatin condensation. In order to stabilize pS^38^Stathmin throughout mitosis, STAT3 serves as an effector of ZIP6/ZIP10 heteromer regulating the expression of both genes.^137,157^ Zinc levels are often higher in cancer tissues than normal tissues, possibly due to the increased demand for tumor growth.^158^ In addition to using zinc chelators to inhibit the proliferative growth of cancer tissues,^78,159–161^ another potential approach is to use ZIP6 or ZIP10-blocking antibodies to hinder mitosis in cancer progression.

#### Maintenance of muscle function

Research indicates that approximately 90% of zinc in the body is found in tissues with slow zinc metabolism, such as skeletal muscle and bone.^162^ Zinc plays a vital role in stabilizing insulin, resulting in a synergistic effect on insulin stimulation of muscle cells.^163,164^ On the other hand, nutritional ZD can hinder skeletal muscle growth, repair, and myoblast differentiation.^165–167^ ZIP7, known as the zinc “gatekeeper”, is localized on the ER and Golgi membrane. It has been extensively studied for its role in skeletal muscle differentiation and the regulation of glucose metabolism^168^ (Fig. 4). The localization of zinc in myoblasts and differentiated myotubes was found to correlate with the changing localization of ZIP7.^169^ Silencing ZIP7 significantly reduces intracellular zinc levels and inhibits Akt phosphorylation, resulting in a decreased number of differentiated cells, even in the presence of extracellular zinc.^170^

Similarly, in myoblasts, knocking down ZIP8 also hampers myotube formation by causing a significant reduction in cellular manganese, iron, zinc, and calcium levels, leading to decreased differentiation and proliferation of myoblasts^171^ (Fig. 4). In comparison, ZIP13 plays crucial roles in the development of bone, tooth, and connective tissue. Mutations in ZIP13 have been linked to the spondylocheiro dysplastic form of Ehlers-Danlos syndrome (SCD-EDS),^172,173^ characterized by abnormalities in hard and connective tissues. ZIP13 knockout mice exhibit delayed growth and skeletal and connective tissue abnormalities, mirroring the phenotypes observed in SCD-EDS patients.^173^

Furthermore, zinc transporters play a direct role in regulating calcium channels, modulating calcium signaling, and subsequently influencing muscle contraction (Fig. 4). For instance, the interaction of ZnT1 with LTCCs enables zinc entry from the extracellular space into the cell membrane, thereby contributing to calcium signaling involved in excitation-contraction coupling in skeletal muscle. Additionally, ZnT1 directly inhibits the activity of L-type calcium channels by binding directly to the β-subunit, Ca_v_β.^151^ ZIP7 and ZnT7 are involved in regulating the release of zinc into the sarcoplasmic reticulum (SR) in skeletal muscle. Intracellular zinc can then modulate ryanodine receptor (RyR)-mediated calcium release from the SR. Notably, the cytoplasmic C-terminal tail of ZnT1 alone can inhibit the channel, suggesting that the inhibition of L-type calcium channels by ZnT1 is independent of zinc channel function.^174^

#### Regulating gastrointestinal (GI) function

The dietary complex releases zinc, which is primarily absorbed by enterocytes in the upper part of the small intestine. The luminal surface cells of the intestinal epithelium originate from intestinal stem cells (iSCs) and comprise various cell types, including enterocytes, goblet cells, enteroendocrine cells, tuft cells, and Paneth cells. These cells express members of both the ZIP and ZnT families involved in zinc transport.^175^ ZIP4 is particularly important for zinc uptake and is closely related to the process. Loss of ZIP4 during embryonic development leads to lethality^176^ (Fig. 4). Previous research has established that ZIP4 is predominantly localized to the apical brush border of enterocytes, facilitating zinc uptake from the intestinal lumen. Furthermore, the expression of ZIP4 is regulated through proteolytic processes that respond to changes in the zinc concentration within enterocytes.^177,178^ Mutations in ZIP4 can lead to acrodermatitis enteropathica, a rare autosomal recessive metabolic disorder characterized by ZD, commonly observed in infants.^179,180^ In the case of ZD, ZIP4 is translocated to the apical surface of the small intestinal epithelial cells. However, when zinc levels are adequate, the mRNA of ZIP4 becomes unstable, and the protein is internalized and quickly degraded.^181^ Intestinal ZnT1 plays a crucial role in zinc acquisition and processing. It is highly expressed in the epithelium of the esophagus, duodenum of the small intestine, and cecum of the large intestine, suggesting its involvement in zinc efflux and absorption into the systemic circulation.^182^ Remarkably, the expression of ZnT1 is influenced by dietary zinc supplementation. Upon zinc supplementation, there is an increase in ZnT1 mRNA expression.^183^ As a result, both ZIP4 and ZnT1 play vital roles in regulating zinc intake.

Zinc plays a vital role in maintaining the homeostasis of intestinal epithelial cells, and its deficiency can lead to alterations in their integrity and function.^184^ Zinc transporters play a significant role in regulating cellular function to support intestinal epithelial homeostasis. Among them, ZnT2 has been proven to be mainly expressed in Paneth cells, which are located within Lieberkühn crypts.^185^ In these specialized secretory cells, ZnT2-mediated zinc absorption into intracellular vesicles is crucial for controlling cytoplasmic zinc levels and cellular function.^185,186^ ZIP4, as mentioned earlier, is important for zinc uptake in the intestine and is essential for the differentiation and maintenance of Paneth cells.^176^ Additionally, ZIP4 also contributes to the proliferation of intestinal epithelial cells.^176^ Mice lacking ZIP4 exhibit disrupted villus integrity, highlighting the significance of ZIP4 in preserving the architecture of the intestinal epithelium. ZIP7, localized to the ER, is also highly expressed in the intestinal crypts.^108,187^ Furthermore, the deletion of ZIP7 greatly enhances the ER stress response of proliferating progenitor cells, leading to apoptosis and disrupting intestinal epithelial cell proliferation and dryness. Indeed, the findings indicate that ZIP7 plays a vital role in promoting both the proliferation and maintenance of stemness in intestinal epithelial cells.^187^

Recent studies have suggested that zinc plays a crucial role in preserving the integrity of mucosal barriers, which is linked to the immunological responses of gastrointestinal diseases in the mucosa^188–190^ (Fig. 4). ZIP14, found at the basolateral membrane of enterocytes along the villus, is particularly abundant in the proximal region of the small intestine.^191^ Deletion of ZIP14 in the intestine has been shown to result in compromised barrier function.^101^ The reason is that ZIP14 maintains the intestinal barrier by stabilizing occludin’s phosphorylation, known as a tight junction protein. Studies have revealed that mice lacking ZIP14 display a disruption in the tight junction complex and increased permeability, potentially due to impaired zinc-dependent activation of ZnR/GPR39. The absence of ZIP14 in mice results in reduced zinc transport into enterocytes, which in turn results in a range of pathologies. These include reduced intestinal barrier function, adiposity, muscle wasting, impaired glucose processing, and skeletal defects that manifest with aging.^191–193^

In the small intestine, ZnT2 assumes a vital function in cytoplasmic zinc buffering, which is essential to Toll-like receptor 4 (TLR4) expression, initiation of pathogen-activated NF-κβ translocation, in addition to the release of cytokine in response to infectious challenges.^185,194^ Furthermore, ZnT2 is indispensable for the development of lysosome biogenesis and bacterial-stimulated autophagy,^195^ facilitating a powerful host defense and resolution machinery against enteric pathogens. In conclusion, this evidence suggests ZnT2 serves as an innovative modulator for mucosal inflammation in colonic cells and plays a crucial role in coping with infectious colitis, opening up possibilities on manipulating ZnT2 as a novel treatment strategy to particular intestinal infections.^194^ ZIP8 is crucial for T cell activation, and recent studies have highlighted its significance in T cell function and innate immunity, which may have important implications in the context of inflammatory bowel disease (IBD).^145,196^ In a study by Li et al., a novel association between Crohn’s disease (CD) and ZIP8 was identified.^197^ Healthy carriers of the ZIP8 variant exhibited changes in intestinal microbiota that partially overlapped with those observed in CD patients. This suggests that disturbances in zinc homeostasis could be linked to ecological imbalances in the gut, potentially contributing to the pathophysiology of CD.

#### Maintaining neuron functions

As a neuromodulator, zinc is crucial in managing diverse synaptic transmissions, such as glutamatergic, GABAergic, and glycinergic.^61,198,199^ In addition, it modulates both short-term and long-term synaptic plasticity, enhances auditory processing, and refines sensory stimulus discrimination.^200–204^ Following physiological activity, vesicular zinc is released and modulates neurotransmission by interfacing with postsynaptic neurotransmitter receptors and activating mZnR/GPR39 signaling.^199,205^

So far, the specific functions of zinc transporters have been described in the brain (Fig. 4). ZnT3, a membrane zinc transporter responsible for concentrating zinc into neuronal presynaptic vesicles and co-released with glutamate upon depolarization, is pivotal in maintaining neuron functions.^206–208^ ZnT3 exhibits predominant expression in the brain, particularly in key regions such as the hippocampus, amygdala, and cerebral cortex.^206^ In various brain areas, including the cerebral cortex, hippocampus, amygdala, and dorsal cochlear nucleus (DCN), the transporter is abundantly present in excitatory neurons, playing a crucial role in channeling zinc into presynaptic vesicles.^209^ Upon synaptic activity, vesicular zinc is released from terminals enriched with ZnT3 and diffuses across the synaptic cleft^61^ to modulate multiple postsynaptic receptors,^199,210^ l including the zinc-sensitive N-methyl-d-aspartate receptor (NMDAR).^61^ The deletion of ZnT3 leads to the suppression of Erk1/2 signaling in MF terminals, resulting in the release of MAPK phosphatase and impairing hippocampus-dependent memory processes.^211^

ZnT1, another zinc transporter, has been suggested to interact with NMDA receptors at synapses.^212^ ZnT1 specifically associates with the C-tail of the NMDAR GluN2A subunit. This ZnT1/GluN2A complex may be influenced by synaptic plasticity, and disruptions in ZnT1 expression led to significant changes in dendritic spine morphology.^61^ The primary targets of the released zinc are NMDARs containing GluN2A, which are responsive to nanomolar levels of extracellular zinc, thereby inhibiting receptor function.^213^ Moreover, ZIP12, exclusively expressed in the CNS, plays a vital role in neuronal differentiation, including tubulin polymerization and neurite extension, by facilitating zinc uptake into the cytosol.^214,215^ Excessive expression of ZIP12 has been observed in schizophrenia.^216^

Additionally, different neuronal populations within the hippocampus express the plasma membrane zinc transporters ZIP1 and ZIP3. While ZIP1 controls the influx of zinc into postsynaptic cells, ZIP3 manages the re-uptake of zinc into dentate granule cells.^217^ SHANK3, a critical scaffold protein in the PSD of excitatory glutamatergic synapses, is sensitive to changes in zinc concentrations. ZIP4 is found in the postsynaptic region and interacts with HOMER1 and SHANK3.^218^

Furthermore, mutations in ZIP8 have been frequently reported in relation to the development of schizophrenia. Genome-wide association studies (GWAS) have indicated that a specific variant of the zinc transporter ZIP8 is significantly linked to the risk of schizophrenia and Parkinson’s disease (PD).^219^ Severe homozygous loss-of-function mutations in ZIP8 lead to a type-II congenital disorder of glycosylation, increasing the risk of schizophrenia.^93,220^ Furthermore, ZIP8 hypofunction may contribute to psychiatric risk by causing glutamate receptor hypofunction and heightened inflammation. As a result, selectively enhancing glutamate function and targeting anti-inflammatory mechanisms could be beneficial for schizophrenia patients with ZIP8 hypofunction.^221,222^ In conclusion, zinc transporters are essential in neuronal cells to maintain neurological function primarily by keeping intracellular zinc ion homeostasis.

#### Involving in glucolipid metabolism

Zinc’s role in insulin crystal formation is widely recognized, with insulin crystallizing in hexamers when two or more zinc atoms are present.^223^ Notably, systemic zinc dysregulation has been demonstrated in both type 1 and type 2 diabetes^224^ (Fig. 4). Pancreatic β-cells, in particular, have elevated zinc concentrations compared to other cell types.^105^ Therefore, if pancreatic β-cells maintain adequate zinc concentrations, the activation of zinc transporters is required.

ZnT8, found in β-islet cells, stands as the most extensively studied zinc transporter involved in insulin formation and secretion.^225–227^ Particularly, the C variant of ZnT8 at single nucleotide polymorphism (SNP) rs13266634 has shown enrichment in individuals with type 2 diabetes, implying its potential influence on diabetes risk.^228^ Notably, polymorphisms in ZnT8 are associated with both type 1 and type 2 diabetes mellitus.^229–231^ Furthermore, ZnT8 autoantibodies are detected in approximately 60–80% of new cases that are clinically confirmed as being affected by type 1 diabetes within the patient population.^232^ When combined with the preexisting detection markers such as protein tyrosine phosphatase IA2, the detection of type 1 diabetes-associated autoimmune responses increases to 98% at the onset.^233^ Interestingly, a distinctive connection between ZnT3 and ZnT8 gene expression in insulin-secreting INS-1E cells has been observed. Conditions that cause an up-regulation of ZnT3 expression, such as high glucose concentration or DEDTC treatment, lead to a down-regulation of ZnT8 expression.^234^ Conversely, knock-down of ZnT3 results in an up-regulation of ZnT8 expression, and vice versa.^235^ Additionally, β-cells express ZIP4, ZIP6, and ZIP7, which play a role in zinc uptake into β-cells, essential for proper insulin packaging,^236–238^ which is required for the proper insulin packaging (Fig. 4).

Currently, the majority of studies have focused on β cells, with only a limited number of studies involving α cells. α cells are responsible for secreting the hormone glucagon, which is essential for the regulation and control of hypoglycemia in the body’s metabolic system, and zinc plays a crucial role as a signal molecule in glucagon secretion. Interestingly, overexpression of ZnT8 in α cells leads to the inhibition of glucagon secretion, which may hold potential benefits for T2D.^239^ Researchers have examined the expression of zinc transporters using fluorescent measurements.^238^ ZIP1 and ZIP14 were found to be the most abundant influx transporters in pancreatic α cells, while ZnT4, ZnT5, and ZnT8 were the dominant efflux transporters.

Besides, zinc has been demonstrated to exert an insulin-mimetic effect on target organs, including adipocytes.^240^ Specifically, it stimulates lipogenesis in fat cells, even in the absence of insulin. Among all zinc transporter functions in lipid metabolism, the role of ZIP13 serving in adipocyte browning has attracted much attention in recent years.^241^ The browning of adipocytes means converting white adipocytes that store energy into beige adipocytes, the energy-consuming brown adipocytes. Fat atrophy is reported in patients with Ehers-Danlos syndrome with mutations in ZIP13 function loss.^173^ Furthermore, ZIP13 has been established as a significant regulator of beige adipocyte differentiation, and it negatively regulates C/EBP-β protein levels. This suggests the physiological importance of the ZIP13-C/EBP-β axis in beige adipocyte biogenesis and thermogenesis, and also highlights its potential in obesity treatment.^242^ Above all, abnormal glucolipid metabolism is not only contributing to the process of diabetes and obesity, but also involved in carcinogenesis,^243,244^ suggesting the unique function of zinc transporters both in clinical and preclinical investigations.

### The physiological role of MTs

#### Involvement in cell proliferation, differentiation, and apoptosis

Numerous studies have demonstrated that MTs regulate zinc, notably in relation to cell cycle regulation and cell proliferation.^245^ MT predominantly resides in the cytoplasm.^246^ Its peak concentration appears during the late G1 and G1/S cell cycle stages.^247^ The nucleus uptake of MTs may be linked to safeguarding cells from DNA damage, apoptosis, and gene transcription through various cell cycle phases.^248,249^

Additionally, MT serves as a donor of zinc to an array of metalloproteins and transcription factors.^250^ DNA-binding proteins featuring zinc finger domains are pivotal in orchestrating DNA transcription processes. The central domain of p53 contains a zinc finger motif, which relies on zinc for structural stability. Apothionein, also known as zinc-free MT, has the ability to remove zinc from p53, leading to a reduction in its transcriptional activity and subsequently suppressing its DNA binding capabilities.^251^ Analogous interactions are observed with the p50 subunit of NF-κB, where MT plays a role in stabilizing the p50-DNA complex. Such interplays have been widely reported for other transcription factors, including Sp1 and TFIIIA.^252,253^ Evidence suggests that MT can modulate cellular activity through the regulation of Zinc. For instance, the protein Bmi1, a member of the Polycomb group (PcG), serves as a crucial epigenetic modulator of stem cell behavior, including aspects like differentiation and self-renewal, throughout both typical maturation and in advanced organ systems.^254^ MT1 plays a facilitating role in this modulation by enhancing resistance, particularly by improving the cellular capacity to combat oxidative stress encountered in their microenvironment, within the satellite cell clusters.^255^ It is worth noting that in DCs treated with zinc chloride (ZnCl_2_), MT1 insufficiency fails to promote a regulatory phenotype specifically aimed at modulating T cell behavior or stimulate the proliferation, such as active growth, of FoxP3^+^ T cells.^256,257^ Besides, MT3’s important contribution to osteoblast differentiation is by counteracting oxidative stress.^258^ Its inhibition of 3T3-L1 adipocyte differentiation is an indirect function, involving the suppression of PPARγ transcriptional activity and a decrease in reactive oxygen species (ROS) levels during early adipogenesis. This indicates that MT3 could be a new target for obesity prevention and treatment.

Furthermore, MTs have also been found to be involved in apoptosis. Recent research has identified XAF1 as a suppressor of MT2A, promoting apoptosis in cellular responses to heavy metals.^259^ XAF1, an exclusive transcriptional target of MTF-1 involved in apoptotic signaling, opposes the survival effects of MT2A, which is also regulated by MTF-1.^259^ Therefore, the induction of XAF1 by heavy metals leads to an apoptotic shift in the stress response by destabilizing MT2A. Additionally, MT mitigates nitrosative damage and cell death caused by angiotensin II (Ang II)-induced NOX.^260^ More specifically, MT2A functions as an anti-apoptotic protein by reducing the expression of caspase-3, caspase-9, caspase-12, and BAX.^261^ In addition, MT2A shields against cardiac failure induced by ER stress by reducing myocardial apoptosis.

#### Maintaining the redox balance

Oxidative stress is defined by an imbalance between oxidants and antioxidants, which arises from the excessive generation of ROS and a decrease in the rate of their elimination by the antioxidant defense system.^262^ The excessive production of ROS, including superoxide, hydrogen peroxide (H_2_O_2_), hydroxyl radicals (·OH), and NADPH-oxidase (NOX), combined with reduced antioxidant capacity, contributes to a pathological imbalance that leads to oxidative stress and inflammation.^121^ Further, this condition would cause cellular and tissue damage, eventually leading to chronic illnesses such as obesity, diabetes, and cancer.^263,264^

Apart from intracellular antioxidants like glutathione (GSH), heme oxygenase-1 (HO-1), superoxide dismutase-1, and nicotinamide adenine dinucleotide phosphate (NAPDH), MTs also serve as a redox buffer by interacting with and scavenging reactive species.^265,266^ Additionally, as a key source of intracellular zinc, MTs play a vital role in the catalytic activation and structural stability of metalloenzymes.^19,267^ Notably, it aids in the structural stability of nitric oxide synthase (NOS),^268^ MMP-9,^269^ and superoxide dismutase (Cu/Zn SOD).^270^ Moreover, MTs become particularly active when the presence of the reduced GSH form is blocked.^271,272^ In this condition, MTs effectively neutralize free radicals using the Zn-MT redox mechanism. MTs contribute to a new pool of thiol in the cell cytosol, mitigating the detrimental effects induced by GSH depletors.^273^ They scavenge ROS through thiol groups present in cysteine residues, displaying stronger antioxidative activity than the majority of well-known antioxidants.^255,274^ Remarkably, MT2A exhibits a 100-fold greater capacity to scavenge free •OH and peroxyl radicals when compared to GSH. In response to oxidative stress, the expressions of MT2A and HO-1 are heightened due to ROS.^275^ MTs also modulate the phosphorylation of ERK and regulate ROS through HO-1.^276^ The potency of MT3 in eliminating ROS has been notably linked to its metal-binding affinity.^277^

MTs’ expression is subject to dynamic regulation by both oxidative stress and cellular zinc levels.^270,278,279^ Under oxidative stress, disulfide bonds are formed, leading to the release of bound metals, particularly zinc, from MTs. While zinc lacks inherent redox capacity, it is regarded as a powerful and crucial antioxidant agent.^279,280^ Several studies have linked cellular zinc depletion to elevated oxidant levels and oxidation parameters. Zinc’s antioxidant properties arise from its direct and indirect interference with target structures.^281^ These functions comprise the induction of MT expression and GSH synthesis, regulation of oxidant production, association with cysteines (alongside release by other oxidants), and modulation of redox signaling. Typically, MT is found in the cytoplasm, but it can also translocate into the nucleus to safeguard DNA from damage and interact with transcription factors, which will be further elaborated on later.^34^

In addition, MT1 and MT2 have differential effects on ROS levels in various organs and tissues. Transcriptionally induced MT1/2 strengthens the liver’s defense system against alcoholic toxicity by reducing ROS and inflammation.^282^ Moreover, IL-22Fc induces MTs in the liver, resulting in decreased hepatic ROS production, stress kinase activation, and inflammatory functions, leading to the amelioration of nonalcoholic steatohepatitis.^283^ MTs play a crucial part in the antioxidative effects of D609, a compound that safeguards RPE cells from oxidative cell death induced by sodium iodate (SI).^284^ Dysregulated MT expression in ascending aortic smooth muscle cells from patients with bicuspid aortic valve (BAV) might lead to an insufficient response to oxidative stress, potentially triggering aneurysm formation.^285^ Recently, MT3 has shown promise for future translational medicine research in osteogenesis due to its effective ROS elimination capabilities.^258^

Besides, the transcription factor MTF-1 enhances cellular protection against oxidative stress, as it responds to alterations in the cell’s redox status.^286^ Specifically, MTF-1 triggers the expression of the *Selenoprotein 1* (*Sepw1*) gene, responsible for encoding an antioxidant GSH-binding protein that effectively scavenges free radicals.^45^ Furthermore, MTF1 can be activated by Sirt6, providing liver protection against alcohol-related liver disease.^282^

#### Orchestrating inflammatory reactions

Extensive research has explored the implications of MTs in inflammation. As mentioned previously, oxidative stress acts as a potent catalyst for releasing inflammatory cytokines,^287^ whereas MT1/2 effectively inhibits the activation of pro-inflammatory cytokines like IL-6, IL-12, and TNF-α.^288^ Studies have demonstrated that bacterial endotoxin LPS acutely induces MT1 expression in various organs, such as liver, heart, kidney, and brain tissues involved in systemic response.^289–291^ In the cellular environment of Histoplasma capsulatum-infected macrophages, the concentrations of MT1 and MT2 expression are regulated by the activation of STAT3 and STAT5 signaling pathways, which are also involved in zinc import, thereby regulating ZIP2.^292^ Liu et al.‘s research revealed that MT2 knockdown increases LPS-induced IL-6 production in endothelial cells,^293^ indicating a protective role against inflammatory responses. Similarly, the absence of MT1/2 significantly exacerbates renal oxidative damage and inflammation induced by intermittent hypoxia, with the Nrf2 signaling pathway implicated.^294^

NF-κB, a crucial inflammation-associated transcription factor, mediates MT1 gene expression.^295,296^ Restoring MT1 expression in cells lacking MT results in the recovery of NF-κB p65 subunit levels, along with a subsequent increase in NF-κB activity related to cellular signaling, and improved protection against apoptosis. These findings indicate that MT1 plays a significant role as a positive regulator of NF-κB activity.^297^ In contrast, MT2A regulates the cell’s inflammatory response by inhibiting NF-κB and endothelial-overexpressed LPS-associated factor-1 (EOLA1).^293^ The increased MT2 expression has demonstrated the ability to reduce NF-κB activity in tumor cells, keloid fibroblasts, and cardiomyocytes.^298–300^ Furthermore, zinc functions as a robust and selective suppressor of IFN-λ3 signaling, resulting in elevated MT levels.^301^

To summarize, MTs possess a wide-ranging and complex ability to regulate inflammatory responses. They serve crucial functions in maintaining a balance by restraining the release of pro-inflammatory cytokines and managing oxidative stress. MTs also influence inflammatory reactions through their impact on essential signal transduction pathways and the expression of diverse transcription factors. The intricate interplay between MTs and crucial elements like zinc forms a complex network of protective mechanisms.

#### Facilitating detoxification of metals

MTs are not only involved in the regulation of zinc homeostasis but also play significant roles in heavy metal detoxification, particularly for cadmium and arsenic.^302,303^ Cadmium, listed as one of the most hazardous substances for human health, accumulates in various organs causing severe oxidative stress and other adverse effects. The protective role of MTs against cadmium toxicity becomes particularly notable here. Exposure to cadmium can displace zinc from MTs and other proteins, leading to an elevation in cytoplasmic zinc levels. This in turn activates MTF-1, inducing MT overexpression.^304^ Interestingly, the cadmium/zinc quotient in MTs determines the level of protection offered to cells against cadmium toxicity. With a lower cadmium/zinc quotient, cells are more protected, while an increased quotient reduces this protection due to the decreased availability of zinc sites for cadmium interaction.^305^ The effectiveness of this protection mechanism was vividly demonstrated in a study conducted among individuals living in a cadmium-contaminated area in China. The study found that individuals with a good zinc status had a notably lower prevalence of renal tubular dysfunction when compared to those who had lower levels of serum and hair zinc.^306^

Exposure to arsenic can result in toxicity, primarily caused by the generation of reactive oxygen intermediates during its redox cycling and metabolic activation.^307^ Zinc acts as a vital safeguard against acute arsenic toxicity through two distinct protective mechanisms: restoration of antioxidant activity and increased expression of MTs.^303^ The enhancement of metal response element (MRE) and antioxidant response element (ARE) activation, facilitated by essential nutrients like zinc, holds the potential to be beneficial in reducing arsenic toxicity. These elements are crucial as they can transcribe the expression of MTs, particularly by minimizing ROS-mediated cytotoxicity, thus adding another layer of protection against arsenic’s harmful effects.^308^ Thus, the multifaceted relationship between MTs and zinc contributes to both heavy metal detoxification and zinc metabolism. Their cooperative function safeguards cellular integrity against the toxicity of heavy metals.

### Cellular zinc metabolism in tumorigenesis

As previously mentioned, there exists a correlation between changes in zinc levels and cancer progression. However, it is essential to acknowledge that the nature of this correlation may vary among various kinds of cancer. Multifaceted effects of zinc in promoting or inhibiting tumor growth underscores this complexity, with distinct mechanisms operating in various cancer types. Recent evidence has been accumulating, suggesting a link between ZD and the development of cancers. Numerous processes are involved in zinc’s anti-tumor activity, encompassing DNA damage and repair, oxygenation, immunity, and the inflammatory process.^45,51,309–311^ Yet it is important to note an increased level of zinc concentration has also allowed for an improved rate of cancer.^312,313^ Since zinc is always characterized by playing a crucial role in growth arrest after the first meiotic division,^153,314^ it also contributes to the proliferation of cancer cells. Furthermore, zinc regulation towards cancer heavily relies on the involvement of zinc transporters. Abnormal expression of these two families is primarily a result of gene dysregulation and translocation from organelles, which result in tumorigenesis mainly through two ways, the regulation of downstream molecular targets and the unsteady state of zinc homeostasis.^315^ Based on this point, we summarized several cancer types whose development is strongly associated with zinc transporters.

#### Breast cancer (BC)

**Fig. 5:**
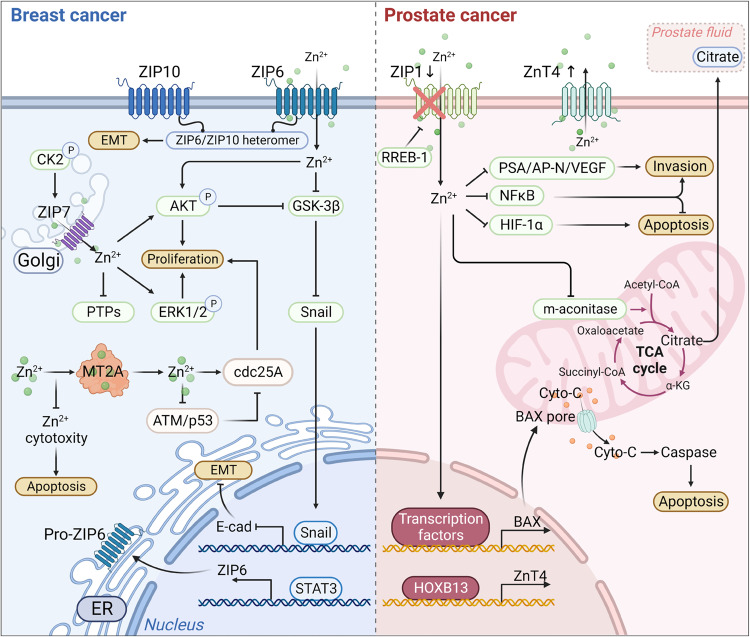
The molecular mechanism of zinc transporters and MTs in BC and prostate cancers. The left figure represents the mechanism of ZIP-mediated proliferation and EMT procession in BC. ZIP7 locates on the endoplasmic reticulum and is highly expressed in tamoxifen-resistant BC cells. After CK2 phosphorylation, ZIP7 was stimulated to transport zinc from intracellular stores, for example, the Golgi apparatus. Subsequently, the increasing zinc concentration can promote proliferation by activating the downstream PTPs, AKT, and ERK1/2 signaling. ZIP6 and ZIP10 locate on the cytomembrane. In addition, ZIP6 is induced by STAT3 and then translocated to the plasma membrane, promoting the accumulation of cellular zinc. The zinc influx caused by ZIP6 and ZIP6/ZIP10 heteromer triggers the AKT pathway and inhibits GSK-3β, finally boosting the EMT process by reducing the nuclear translocation of Snail. MT2A play a dual role in zinc homeostasis and BC cell proliferation. They can chelate zinc ions to reduce zinc cytotoxicity-induced apoptosis, while also releasing zinc ions to promote cancer cell proliferation through cdc25A activation. The figure on the right elucidates the mechanism of zinc transporter involved in prostate cancer. RREB-1 downregulates ZIP1 expression, leading to zinc homeostasis imbalance in prostate cells. ZIP1 downregulation reduced zinc influx, thus degrading the Bax pore expression level, which is the channel for cyto-C releasing into the cytoplasm. Consequently, the apoptosis induced by cyto-C is inhibited. Moreover, decreasing zinc concentration attenuates the inhibition of m-aconitase, which drives citrate oxidization in the TCA cycle. Meanwhile, the inhibitory effect of zinc on the NF-κB signaling pathway was diminished, as well as the inhibitory effect on the expression of HIF-1α, PSA, AP-N, and VEGF, which contributes to the invasion and proliferation. Besides, HOXB13 upregulates the expression of ZnT4 in prostate cancer through transcriptional regulation. EMT epithelial-mesenchymal transition, CK2 casein kinase 2, PTPs protein tyrosine phosphatases, RREB-1 Ras-responsive element binding protein 1, m- aconitase mitochondrial aconitase, cyro-C cytochrome C, PSA prostate-specific antigen, AP-N activity of urokinase-type plasminogen activator and aminopeptidase N, VEGF vascular endothelial growth factor, TCA tricarboxylic acid

Studies have reported that BCs, along with malignant cell lines, exhibit a higher accumulation of zinc in contrast to normal mammary epithelium.^316,317^ Moreover, the degree of zinc accumulation has been linked to cancer progression and malignancy.^318,319^ ZIP6 (also known as LIV-1), was initially recognized as an estrogen-mediated gene since 1988.^134,320,321^ It is observed to be upregulated in estrogen receptor-positive breast cancers and shows a positive correlation with estrogen receptor status. During gastrulation in zebrafish, zip6 is transactivated by STAT3. Elevated expression of zip6 results in nuclear retention of Snail, which is also known to be a zinc-finger transcription factor, which subsequently represses the expression of E-cadherin, resulting in cell migration^322^ (Fig. 5). Indeed, E-cadherin performs its function as a calcium-induced TM glycoprotein, with its decreased expression linked to BC metastasis.^323,324^ Taylor’ research observed a positive association between STAT3 and ZIP6 in breast cancer samples.^320^ Furthermore, the induction of ZIP6 expression by STAT3 induces the translocation of ZIP6 to the plasma membrane and facilitates zinc influx, which is triggered by N-terminal cleavage.^157^ Consequently, the zinc influx activates the zinc influx/GSK-3β inhibition/Snail activation/E-cadherin loss pathway, resulting in cell rounding and detachment (Fig. 5).

However, despite the above discoveries, a solid link of ZIP6 to lymph node metastasis has not yet been entirely determined. There is evidence that ZIP6 is negatively correlated with EMT.^325^ E-cadherin is downregulated in the condition of ZIP6 silencing.^326^ In BC cells, exposure to high glucose results in a notable elevation of intracellular zinc levels, and it also leads to decreased mRNA expression of ZIP6 in the context of hypoxia. This downregulation of ZIP6 is associated with increased cell viability and reduced E-cadherin expression.^327^ Hypoxia, which arises due to the aggressive proliferation of tumor cells, has previously been shown to trigger BC cells to undergo EMT, thereby promoting cell survival and malignant progression.^328,329^ Similarly, the knockdown of ZIP6 blocks the balance of intracellular zinc levels, resulting in more tolerant cells in hypoxic environments.^321^ Furthermore, some evidence suggests ZIP6 is associated with a more favorable prognosis. An illustration of this is that ZIP6 serves as a biological marker for estrogen receptor-positive luminal-type-A breast cancer, which is a molecular subtype associated with a more favorable prognosis.^330–332^

Among the ZIP zinc transporter family, ZIP10 shows the highest similarity to ZIP6, sharing 43.5% sequence identity, which implies that they likely possess comparable roles in the regulation of cell migration.^154,333^ As an indicator of metastasis and aggressiveness in cancer progression, ZIP10’s clinical relevance extends to its correlation of estrogen receptor ERBB3 and STAT3 among BC cases,^320,334,335^ like the previously mentioned ZIP6. In mitosis, ZIP6/ZIP10 heteromer-induced zinc influx into cells leads to the formation of pS^727^STAT3 from pY^705^STAT3. PY^705^STAT3 serves as a transcriptionally promoted form of the protein,^336,337^ impelling numerous malignant cancer features, such as EMT in HER2-positive BCs.^338^ Chandler et al. discovered that the elevated presence of ZIP10 as well as the reduction in ZIP4, ZIP7, and ZIP11 were consistent mechanisms linked to zinc overaccumulation in the cells of malignant mammary glands.^39^

Furthermore, the expression of ZIP7 has been demonstrated to be remarkably upregulated in BC cells.^339,340^ ZIP7 functions as a zinc importer, moving zinc from intracellular stores (i.e., ER, Golgi) to the cytoplasm upon stimulation by the phosphorylation of CK2^168^ (Fig. 5). The upregulated expression of ZIP7 facilitates the proliferation and aggression of tamoxifen-resistant MCF-7 cells by activating epithelial growth factor receptor (EGFR), insulin-like growth factor receptor 1 (IGF1R), and tyrosine kinase Src.^339^ Activated ZIP7 is essential to the proliferation of drug-resistant estrogen receptor-positive BC.^340^ Additionally, it is of great importance to note that ZIP7 plays a vital role in ferroptosis, which may establish a connection between ferroptosis susceptibility and treatment-resistant cells, as described in reference.^159^ Mechanistically, ZIP7 overexpression induces zinc mobilization from the ER and Golgi,^341^ triggering tyrosine kinase signaling as well as enhancing the aggressiveness of MCF7 cells^148,339^ (Fig. 5). Besides, ZIP13 expression and subsequent mobilization of zinc from the ER/Golgi are essential for stimulating BMP/TGF-β signaling in connective tissue.^173^ Overexpression of ZnT2 has resulted in cell cycle shifts, increased apoptosis, and decreased proliferation and invasion capabilities within MDA-MB-231 cells.^39^ To summarize, being a risk factor for BC, zinc ions are regulated by ZIPs and ZnTs. Unlike ZnTs, the transporter proteins responsible for zinc inward flow, ZIPs, appear to be oncogenes in BC.

Indeed, there is evidence of mechanistic heterogeneity in the function of zinc transporters across different subtypes of BC. A notable association has been found between ZIP6 mRNA expression and improved overall survival (OS) among the whole cohort, the same as patients with luminal A and HER2-positive tumors.^342^ Conversely, in luminal B and triple-negative BC (TNBC) subtypes, patients with high levels of ZIP6 expression showed worse OS. Besides, within the context of this heterogeneity, ZIP4 transporter plays a distinct role, particularly in TNBC. The upregulated ZIP4 expression results in enhanced zinc influx and promotes tumorigenicity in TNBC.^343^ Interestingly, the intracellular zinc concentration in the BrM2 cell line, which metastasized to brain tissue, was found to be twice as high as that in the TNBC cell line MDA-MB231. Additionally, ZIP8, ZIP9, and ZIP13 have been demonstrated to be upregulated in BrM2 cells. The correlation between intracellular zinc concentration and BC cell metastatic potential is implied.

However, excess zinc accumulation typically triggers apoptosis, necessitating mechanisms in malignant breast cells to protect themselves from zinc-induced cell death. MTs serve as buffers for cellular zinc and shield cells from zinc toxicity. Breast tumors are known to hyper-accumulate zinc, with tissue biopsies of invasive ductal carcinoma overexpressing MTs in up to 88% of cases,^344^ reflecting aberrant zinc accumulation and associated with poor prognosis. Furthermore, MT expression inversely correlates with estrogen receptor expression, indicating an important protective role for MT overexpression in highly invasive and poorly differentiated breast carcinoma. Specifically, TCGA data showed that patients with estrogen receptor α-positive BC had reduced concentrations of MT1 genes.^345^ Nevertheless, it should be noted that not all malignant breast cells express MTs, implying the presence of alternative mechanisms to prevent zinc cytotoxicity. ZnT2, similar to MTs, exhibits zinc-responsive expression due to MREs in its promoter, as previously mentioned.^122^ The overexpression of ZnT2 has been observed in MT-null BC cells (T47D). It is positively correlated with zinc accumulation, thereby conferring a protective effect against excess zinc-induced cytotoxicity.^346^

Additionally, MT overexpression is primarily observed in the invasive ductal carcinoma subtype of BC and is associated with p53 inhibition and resistance to apoptosis.^122,344^ As previously mentioned, apo-MT was able to eliminate zinc from p53, and reduced the subsequent transcriptional activity, yet it was incapable of binding to DNA.^251^ Moreover, MTs can influence BC growth through cell cycle effects. In BC cells, suppression of MT2A results in an upregulation of ataxia telangiectasia-mutated (ATM) expression and a concurrent decrease in cell division cycle 25 A (Cdc25A) levels.,^347^ which is known as playing a pivotal character in facilitating the cell cycle transition from G1 to S phase. Interestingly, cdc25c, which originated from the cdc25 protein family as well, has been characterized as a zinc-binding metalloprotein. Its role involves dephosphorylating and activating the Cyclin B/cdk1 complex, which subsequently governs the initiation and advancement of mitosis.^348^ On the other hand, p53 is identified as the substrate related to ATM coping with DNA damage.^349^ The subsequent induction of CDK inhibitor p21 CIP1/WAF1 transcriptional activity results in a G1-growth arrest.^350^ Thus, MT2A may serve as a zinc donor and plausibly promote cell cycle progression through the ATM-cdc25A-dependent pathway in BC.

To sum up, zinc metabolism is critical to the pathogenesis of BC, with zinc transporters, particularly ZIP6, ZIP7, and ZIP10, along with MTs and ZnT2, having profound effects on cellular processes like cell migration, cell viability, and apoptosis. These molecules not only impact zinc homeostasis within the cancer cells but also modulate important signaling pathways and cellular responses to hypoxic environments, thereby influencing the progression and outcome of the disease.

#### Prostate cancer

**Fig. 6:**
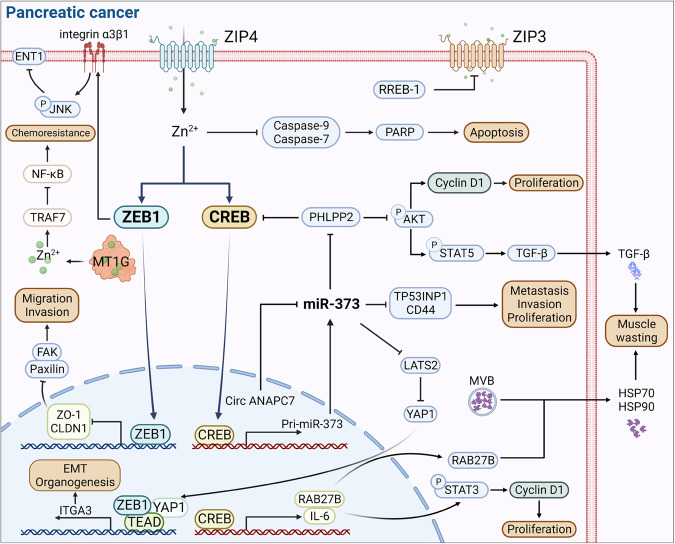
The molecular mechanism of zinc transporters and MTs in PC. ZIP4 promotes PC carcinogenesis mainly through two transcription factors, CREB and ZEB1. ZEB1 promotes the procession of EMT by suppressing the expression of ZO-1 and CLDN1 and inducing the transcription of ITGA3. Moreover, the ZEB1 induces integrin α3β1 to phosphorylate JNK and ultimately blocks ENT1, a gemcitabine transporter, which results in chemoresistance. Besides, cellular zinc released by MT1G inhibits NF-κB, suppressing PC chemoresistance. CREB transcripts miR-373 to increase metastasis, invasion, and proliferation by activating the Hippo pathway yet inhibiting the expression of *TP53INP1* and *CD44*. Besides, PHLPP2, inhibited by miR-373, forms a malignant cycle through the suppression of CREB. However, the small molecule, circ ANAPC7, can block miR-373. As a target for PHLPP2 dephosphorylation, AKT increases the proliferation by upregulating cyclin D1 and promotes muscle wasting by phosphorylating STAT5. Another CREB-mediated downstream promoting muscle wasting is RAB27B. Mechanically, RAB27B promotes the release of HSP70 and HSP90 from MVB. Additionally, the CREB-mediated IL-6/STAT3/cyclin D1 pathway leads to proliferation in PC. ZIP4 could restrain apoptosis by inhibiting the activity of caspase9 and caspase7. The expression of ZIP3 is reduced by RREB-1. ZO-1, zonula occludens-1; ITGA3, integrin subunit alpha 3; JNK, c-Jun N-terminal kinase; MVB, multivesicular body; EMT, epithelial-mesenchymal transition

Of all the soft tissues in human bodies, normal and hyperplastic prostate tissues have the highest concentrations of zinc accumulation.^351^ On the other hand, zinc concentrations detected in prostate cancer were greatly reduced.^352^ The peripheral zone, which is found to serve as the origin of prostate cancer, is responsible for secreting prostatic fluid. An essential and distinctive component of this fluid is the remarkably high concentration of citrate.^353–355^ Traditionally, citrate is oxidized in the tricarboxylic acid (TCA) cycle, while high cellular zinc levels in normal prostate cells prevent this process by inhibiting the activity of mitochondrial aconitase (m-aconitase)^356^ (Fig. 5). Furthermore, to preserve normal prostate function, physiological zinc levels induce apoptosis through various mechanisms in prostate cells. These include upregulating the Bax/Bcl-2 ratio in the mitochondria,^357^ inducing HIF-1α degradation,^358^ and involving with NF-κB pathway^359^ (Fig. 6). Besides, zinc is also involved in the inhibition of invasion and adhesion in malignant prostate cancer cell through several ways: strongly prevents the enzymatic activity of prostate-specific antigen (PSA) and suppresses the invasion of LNCaP cells,^360^ reduces the expression of vascular endothelial growth factor (VEGF),^361^ interleukin (IL)-6, IL-8, matrix metalloproteinase-9 (MMP9), intercellular adhesion molecule-1 (ICAM1), diminished the activity of urokinase-type plasminogen activator and aminopeptidase N (AP-N)^362^ (Fig. 5). Unfortunately, prostate cancer cells have significantly lower zinc levels, and hence they are unable to inhibit m-aconitase activity, ultimately resulting in the inability to obtain normal prostate fluid with citrate in tissue.^363^ Also, m-aconitase activity can contribute to the proliferation and migration of prostate cancer cells.^364^ Indeed, the low zinc concentration in malignant cells possesses mechanisms such as ZIP downregulation and ZnT upregulation.

ZIP1 predominantly localizes at the basolateral membrane. Both normal and hyperplastic prostate glandular epithelial cells have in situ expression of ZIP1, where it transports zinc from the plasma into the cell.^365,366^ In most cases, it plays a predominant role in zinc accumulation in benign prostatic hyperplastic epithelial cells. In contrast, ZIP1 is downregulated in malignant cells, resulting in the inability to accumulate zinc.^367–369^ Therefore, prostate cancer can be characterized as a ZIP1-deficient tumor.^370^ The expression of ZIP1 and ZIP2 detected by RT-in situ-PCR was lower in African Americans’ prostate epithelial cells than in Caucasian men, which could be involved in the higher susceptibility of African-Americans to prostate cancer.^367^ Interestingly, overexpression of ZIP1 can sensitize the tumorigenic prostate epithelial cells (RWPE2) to tumor necrosis factor (TNF)-related apoptosis-inducing ligand (TRAIL)-mediated apoptosis.^371^ It was shown that the core promoter regions, contributing to the regulation of ZIP1 expression, are modulated by SP1 as well as CREB.^372^ RREB-1, the downstream of ERK in the Ras/Raf/MAPK pathway, was upregulated in prostate cancer progression.^373–375^ The inhibition of ZIP1 expression in prostate cancer implicates the mobilization of RREB-1, which could become one of the possibilities for the downregulated expression of the zinc transporter in malignant prostate disease^376^ (Fig. 5). Besides, ZIP1-mediated rapid increase of zinc levels seems to be androgen-dependent.^377^ Furthermore, by acting as an androgen cell membrane receptor, ZIP9 facilitates the mechanism of testosterone-dependent apoptosis in prostate carcinoma.^378,379^

Unlike ZIP1, ZIP2 and ZIP3 are hardly localized to the basolateral membrane, both of which are mainly constrained to the apical membrane of the prostate tissue.^380^ Studies on cell lines suggest that the functional role of ZIP2 and ZIP3 is to transport or reabsorb zinc from prostatic fluid back to the epithelium,^14,381^ rather than accumulating cellular zinc from the blood circulation, which is the primary function of ZIP1.^382^ Human prostate tissue sections examined by immunohistochemistry examination show significantly reduced regulation of ZIP2 and ZIP3 in adenocarcinoma glands, leading to dysfunction in accumulating zinc.^371,380,383^ Thus, it is reasonable to propose that ZIP1, ZIP2, and ZIP3, all of which belong to the ZIP family, function as tumor suppressor genes in prostate carcinogenesis.

Regarding the ZnT transporter family, ZnT4 is five times higher in prostate cancer as measured in normal tissues.^111^ Furthermore, ZnT4, as well as ZnT10, is highly induced by the HOXB13.^384^ The introduction of exogenous HOXB13 decreases intracellular zinc levels in prostate cancer cells and activates NF-κB signaling, which promotes prostate cancer invasion. In addition, ZnT4 mRNA was found to be overexpressed in tumor samples acquired through radical prostatectomy versus normal tissues.^385^ Interestingly, ZnT5 was also expressed at high levels in human prostate tissue.^386^ Further study of the mechanistic impact of altered zinc transporter expression levels on prostate carcinogenesis has important implications for clinical treatment.

Additionally, studies investigating the relevance between MT expression and pathological/malignant conditions are severely limited in the prostate, and the regulatory mechanisms of zinc on MTs expression in prostate cells remain unclear. MT1/2 downregulation has been observed in benign prostatic hyperplasia (BPH), PC-3 cells, and malignant tissues of the human prostate. MT1/2 expression is notably enhanced by zinc therapy in both PC-3 and BPH cells, coincident with the restoration of intracellular zinc concentrations. Specifically, in BPH cells, MT3, acting as a growth inhibitory agent, was identified, and its levels were elevated by zinc. Furthermore, the expression of MT3 serves as a distinctive feature exclusively found in BPH cells.^387^ MT1h, one of the components of the MT1 family, is commonly decreased in prostate cancer. The heavy methylation of its promoter has been observed. MT1h exerts its role as a tumor suppressor by activating euchromatin histone methyltransferase 1 (EHMT1), which leads to histone methylation and potentially suppresses gene expression.^388^

#### Pancreatic cancer (PC)

Despite tremendous research efforts in the past few years, PC remains one of the most devastating diseases and has the highest fatality rate among all cancers.^389^ Accumulating evidence indicates a strong correlation between zinc transporters and PC growth and progression.^75,312,390–392^ However, the zinc levels and the molecular mechanisms through which zinc transporters regulate cancer growth in PC are not yet fully understood. Therefore, it is essential to study the effects of zinc transporters in PC carcinogenesis.

Overexpression of ZIP4 is widely described in human PC tissues and cell lines, contributing to tumor growth.^75,393–399^ Obviously, the potential role by which ZIP4 is involved in PC growth and migration may be multifaceted. Knocking out ZIP4 is able to suppress the proliferation of PC through reducing cyclin D1 expression,^393^ which serves as the downstream target of CREB/miR-373/PHLPP2 and CREB/IL-6/STAT3 pathway. Both pathways are activated by the overexpression of ZIP4, leading to PC cell proliferation^400^ (Fig. 6). ZIP4 contributes to the mediation of metastasis in addition to the proliferation of PC cells. ZEB1 is the most critical EMT-associated transcription factor in PC, promoting stemness, invasion, and metastasis of PC.^401^ Significantly, ZIP4 induces the expression of ZEB1, which mechanically is through phosphorylated STAT3.^395^ Another report suggested that ZIP4 activates PC migration and invasion by mediating ZEB1 inhibition of ZO-1 and Claudin-1 expression^394^ (Fig. 6). Additionally, ZIP4 is able to induce the expression of YAP1 by stimulating a miR-373-LATS2 pathway in PC, promoting organ formation and cell adhesion through the increasing expression of ITGA3.^74^ Notably, the upregulation of ZEB1 inhibited expression of the gemcitabine transporter via ITGA3/ITGB1/α3β1 signaling and c- JNK pathway, which leads to chemoresistance both in vitro and in vivo.^395^ Moreover, ZIP4 has a notable role in PC-related cachexia, where it facilitates the release of HSP70 and HSP90 via extracellular vesicles, thereby stimulating muscle atrophy.^75^ Whereas the CircANAPC7 inhibited ZIP4/miR-373 mediated muscle wasting partially through STAT5/TGFβ signaling in PC.^400^ These findings suggest that ZIP4 might serve as a potential PC diagnosis and therapy target (Fig. 6).

It could infer that aberrant overexpression of ZIP4 elevates zinc concentrations in PC cells. Using the nude mice model with subcutaneous xenograft, a study found that 80% more zinc was detected in the tumors implanted with ZIP4 stably overexpressed MIA-ZIP4 cells compared with the normal group.^393^ However, clinical and preclinical indications disclose that zinc is persistently and significantly reduced in the early stage of PC compared with the normal or benign pancreas tissues, which is an essential malignant event.^402^ Indeed, the reduction in zinc levels in pancreatic intraepithelial neoplasia (PanIN) lesions and malignancy is attributed to the downregulation of Ras responsive element binding protein 1 (RREB-1) and the silencing of ZIP3.^380,390,402^ Another study has proved that PC cells are vulnerable to high zinc concentrations. The exposure of PC cells to physiological concentrations of zinc (0.01–0.5 mM) can lead to cytotoxic cell death, which is characterized by up-regulation of the zinc transporter ZnT1 gene expression.^312^ Another study revealed that higher levels of zinc chloride (>50 μM) significantly reduced the proliferation of MIA-ZIP4 cells, suggesting that zinc activated the proliferation of PC cells only at comparatively low concentrations.^393^ Besides, zinc provided by MT may be working with transcription factors. Research has shown that MT1G plays a crucial role as a tumor suppressor in pancreatic cancer stem cells. The downregulation of MT1G, caused by hypermethylation of its promoter, is associated with the maintenance of pancreatic cancer stemness. Mechanistically, MT1G exerts a negative regulatory effect on NF-κB signaling and facilitates the degradation of the NF-κB p65 subunit by upregulating the expression of E3 ligase TRAF7, consequently suppressing PDAC stemness.^403^

Apparently, zinc is essential for cellular function, growth, reproduction, and metabolism. Thus, normal cells have evolved homeostatic mechanisms to maintain their normal required zinc levels and prevent the potential adverse effects of excessive zinc concentrations. However, the malignant cell has lost these normal protective conditions. PC cells require excess zinc to support proliferation and, on the other hand, avoid the adverse effects of zinc through other regulatory mechanisms.

#### Colorectal cancer (CRC)

Notably, a meta-analysis of human studies indicated that higher zinc intake was inversely associated with the overall risk of digestive tract cancers, especially for CRC.^404^ It has been reported that zinc can inhibit the proliferation of colon cancer cells by arresting the cell cycle in the G2/M phase and disrupting the microtubule stability of cell-cell communication.^405^ Hence, zinc transporters could be involved in GI disorders.

By bioinformatic analysis of microarray data in the GEO database, it has been identified that *ZnT10* is one of the ten recommended candidate genes associated with CRC.^406^ Consistently, a recent study reported ZnT10 as a methylation marker in the CRC, and the methylation epigenotype significantly correlated with KRAS and BRAF mutation in CRC.^407^ In contrast, reduced expression of ZnT10 is associated with aggressive tumor phenotypes and poor patient outcomes in CRC.^408^ ZnT10 acts as a competitive endogenous RNA for miR-21c to upregulate tumor suppressor gene APC expression, thus inhibiting CRC progression and metastasis.^408^

**Fig. 7:**
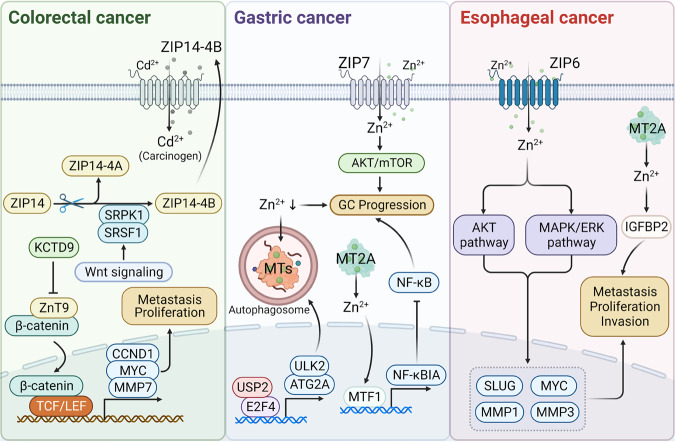
The molecular mechanism of zinc transporters and metallothioneins in CRC, GC, and ESCC. In CRC, the binding of ZnT9 and β-catenin triggers the transcription of *CCND1*, *MYC*, and *MMP7*, resulting in proliferation and migration. KCTD9 can replace the binding of ZnT9 and β-catenin. Moreover, ZIP14 contains two alternative splicings, ZIP14-4A and ZIP14-4B. ZIP14-4B is upregulated by SRPK1 and SRSF1, two downstream targets of Wnt signaling, leading to increased Cd^2+^ uptake. Concerning the GC microenvironment, ZIP7 is the upstream target of the AKT/mTOR signaling pathway. In GC, autophagic degradation of MT1E, MT1M, and MT1X initiated by USP2-E2F4 interaction leads to increased intracellular zinc storage vesicles, promoting GC cell growth. In contrast, MT2A inhibits NF-κB by releasing cellular zinc and thus ultimately suppresses GC cell proliferation. As for ESCC, ZIP6 activates PI3K/AKT and MAPK/ERK signaling pathways, which leads to the overexpression of downstream oncogenes such as *MMP1*, *MMP3*, *MYC*, and *SLUG*. Meanwhile, the cellular zinc released by MT2A promotes the oncogenic function of IGFBP2. NF-κBIA, NF-κB inhibitor alpha; IGFBP2, insulin-like growth factor binding protein 2

Additionally, ZnT9 is the coactivator of β-catenin-mediated gene transcription,^409,410^ which serves as the critical event in the Wnt signaling pathway and the development and progression of colon cancer.^411^ Notably, the binding of ZnT9 and β-catenin can be competitively replaced by KCTD9, a tumor suppress gene which is negatively correlated with the clinical CRC stage, thus substantially inhibiting the transcription of downstream oncogenes, including *MYC*, *CCND1*, and *MMP7*^409^ (Fig. 7). In fact, ZIP7 also plays a crucial role in intestinal epithelial self-renewal.^187^ Colorectal tumors have higher expression levels of ZIP7 than normal colon tissues.^412^ It was demonstrated that the knockdown of ZIP7 induced G2/M cell cycle arrest and promoted apoptosis in colorectal cancer cells.^413^ Furthermore, the downregulation of ZIP7 promoted the cleavage of PARP, enhanced the expression of Bad, Caspase-9, and cleaved-Caspase-3, and suppressed Bcl-2 expression in CRC.^413^

Alternative splicing is a critical step in generating protein diversity, and its misregulation has been observed in carcinogenesis.^414–416^ Notably, alternative splicing of ZIP14 was found to be regulated by the Wnt pathway in CRC, most likely through the regulation of SRPK1 and SRSF1^417^ (Fig. 7). ZIP14 contains two mutually exclusive exons, 4 A and 4B, and the ratio of exon 4 A/4B was significantly reduced in adenomas and cancers, which may be used as a tumor marker for identifying CRC and precancerous lesions. Specifically, the exon 4B isoform of ZIP14 is found to have an eightfold higher affinity for Cd^2+^ than the exon 4 A isoform, which is known as a potent carcinogen.^99^ Moreover, Cd^2+^ has been found to influence several cellular processes, including apoptosis, differentiation, and cell growth, especially the inhibition of DNA mismatch repair,^418,419^ thus setting off CRC carcinogenesis.

Beyond the roles of ZIP and ZnT transporters in CRC, our review extends to proteins such as MTs that regulate cellular zinc metabolism. Intriguingly, these MTs often act as tumor suppressor genes in CRC. A notable correlation between low MT1B, MT1H, or MT1L expression and an increased risk of adverse outcomes was identified.^420^ Additionally, a distinct four-gene model, consisting of MT1F, MT1G, MT1L, and MT1X, effectively predicted survival and CRC prognosis. It has been reported that zinc potently enhances MT expression and is cytotoxic to cancer cells.^421^ MT2A expression decreased in colorectal cancer and was linked to the patient’s tumor M stage.^422,423^ The present research has mechanistically illustrated that MT2A upregulation promoted the expression of phosphorylated MST1, LATS2, and YAP1, which consequently inhibited the Hippo signaling pathway and controlled CRC cell proliferation and liver metastasis.^422^ However, it is unclear whether the role of MT in controlling the MST1/LATS2/YAP1 signaling pathway depends on its regulation of zinc. Thus, the role of zinc and its regulatory mechanism in CRC requires further in-depth investigation.

#### Gastric cancer (GC)

In GC studies, the relationship between zinc intake and GC is contradictory. On the one hand, a large number of studies point out that lower zinc intake may increase the risk of GC.^424–426^ For example, Cixian and Linxian are one of the higher-risk areas for upper GI cancer both in China and worldwide, where individuals have a zinc intake below the recommended daily allowance and higher incidence and mortality rates of GC than that of other regions.^427–429^ However, a meta-analysis revealed that zinc intake was significantly associated with GC risk in Asia but not in America and Europe.^404^ The heterogeneity in the results of zinc intake associated with GC risk may be due to the differences in the expression background of zinc transporters.

Multiple bioinformatic approaches revealed that high expression of five genes (*ZnT1*, *5-7*, and *9*) was significantly correlated with better overall survival (OS), first progression survival (FPS), and post-progression survival (PPS), while upregulated *ZnT2-4*, *8*, and *10* expressions was markedly associated with poor OS, FP, and PPS.^430^ In addition, ssGSEA analysis indicated that *SLC30* family genes were closely associated with the infiltration of immune cells, indicating that the ZnTs induced tumorigenesis partly because of immune infiltration.^430^

In the GTEx and TCGA datasets, ZIP10 was highly expressed at the mRNA level in malignant GC cells compared to normal and adjacent non-tumor samples.^431^ A previous study has demonstrated that ZIP10 expression was correlated with STAT activation in B cell lymphoma samples.^137^ In GC, the novel natural product inhibitor of STAT3 termed XYA-2 might exert its anticancer activity by synergistically inhibiting the expression of MYC and ZIP10, two downstream genes of STAT3 in vitro and in vivo.^431^ Meanwhile, ZIP6, another downstream target of STAT3, is involved in cancer development by forming a heterodimer with ZIP10.^157^ Besides, the ZIP7 mRNA level was increased in both GC tissues and cell lines, which boosted cell proliferation and migration, while inhibiting apoptosis in GC.^432^ Specifically, ZIP7 was negatively regulated by miR-139-5p and positively regulated GC development through Akt/mTOR signaling pathway, suggesting that ZIP7 may be a candidate target gene for GC treatment (Fig. 7).

Alarmingly, reduced expression of MT1 or MT2 has been observed in GC, a pattern correlated with worse prognoses.^433^ There has been an observed decrease in MT2A and myeloid zinc-finger 1 (MZF1) expression in clinical specimens that are undergoing malignant transformation of the stomach.^434^ Intriguingly, an important role played by zinc accumulation in controlling cancer through autophagy flux has been reported.^435^ Autophagic degradation of MT1E, MT1M, and MT1X, initiated by E2F4 in GC, leads to an increase in zinc-stored vesicles within autophagosomes. This, in turn, lowers the levels of free intracellular zinc and facilitates the growth and invasion of GC cells. These findings offer a novel insight into how autophagy modulates zinc homeostasis in cancer cells.^48^ In line with this, recent evidence has indicated that MT1M has the ability to dampen the malignancy and stem cell-like characteristics of GC by inhibiting GLI1, a component of the Hedgehog signaling pathway, known for its numerous zinc finger domains.^436^ Besides, the MT1 gene cluster has been found to be hypermethylated in EBVaGC, suggesting redundant anti-EBV roles among various MT1 genes.^437^ MT1 proteins provide cellular protection against OS via their antioxidant properties,^438^ which account for their anti-EBV functions.

Furthermore, in human GC cell lines and primary tumors, the transcription factor MZF1 has been found to be epigenetically silenced, a finding associated with MT2A. MZF1 serves to deter gastric carcinogenesis by associating with MT2A to bind to the NFKBIA promoter (Fig. 7). Notably, this tumor-suppressive effect can be stimulated by diallyl trisulfide (DATS), a compound derived from garlic known to thwart the progression of GC.^439^ In keeping with the ability of zinc to inhibit NF-kB activation in cancer cells,^440–442^ zinc chelation likely plays a part in the anti-GC activity of the MT2A/MZF1–NF-kB pathway mediated by DATS. MT2A simultaneously controls zinc-binding proteins by adding or removing zinc and is transcriptionally inducible by these proteins to target its promoter region, which contains numerous regulatory elements, such as the MRE.^434^ Therefore, the diminished expression of MZF1/MT2A significantly associates with the malignancy of GC and poor patient outcomes. Additionally, MT2A hinders cell growth via apoptosis and G2/M arrest, negatively influencing the NF-κB pathway through upregulation of IκB-α and downregulation of p-IκB-α and cyclin D1 expression.^298^ ApoMT (metal-free MT) has been identified as a potential agent for extracting zinc from NF-κB, thereby rendering the NF-κB-mediated transcriptional activity inactive due to zinc chelation.^443^ In conclusion, targeting GC by interfering with zinc metabolism appears to be a viable approach (Fig. 7).

#### Esophageal squamous cell carcinoma (ESCC)

Another essential type of digestive tract tumor is ESCC. Actually, ZD in dietary potentiates the effects of specific nitrosamines that act as esophageal carcinogens in rodents.^444^ A study using x-ray fluorescence to measure zinc concentrations in tissues demonstrated that zinc concentration is inversely associated with the risk of incident ESCC.^445^ Zinc replenishment rapidly induced apoptosis in esophageal epithelial cells and thereby substantially reduced the development of ESCC.^446^

However, ZIPs, the proteins that translocate Zinc into cells, are associated with ESCC. Immunohistochemical staining of ESCC tissues showed that higher expression of ZIP6 predicted unfavorable prognosis in individuals with advanced ESCC.^447^ ZIP6 overexpression is an “early” or “intermediate” event in the ESCC malignant progression, indicating that ZIP6 could serve as an early detector of high-risk subjects and prognostic biomarker.^448^ Cheng et al. revealed that overexpression of ZIP6 or elevated intracellular zinc levels in cancer cells substantially activated the PI3K/AKT and MAPK/ERK signaling, which upregulated downstream oncogenes such as *MMP1*, *MMP3*, *MYC*, and *SLUG*.^449^ This up-regulation of these molecules may be the underlying mechanism for the aggressive phenotypes of ESCC with ZIP6 overexpression (Fig. 7).

Similarly, studies suggested that ZIP5 protein and mRNA expression was highest in ESCC, intermediate in paraneoplastic tumors, and lowest in normal tissue.^450^ Kumar et al. found that the dysregulation of zinc homeostasis in esophageal tumorigenesis is mainly reflected in the upregulation of ZIP5 and the downregulation of the zinc metabolism protein MT1G using cDNA microarray.^451^ Besides, the downregulation of ZIP5 decreased the expression of COX2 and increased the expression of E-cadherin in the KYSE170K xenografts.^452^ COX2 is an essential molecular basis for cancer progression, which promotes the proliferation and invasive ability of tumors and inhibits cancer cell apoptosis.^453^ Collectively, knocking down ZIP5 by small interfering RNA might be a novel therapeutic strategy for ESCC with ZIP5 overexpression. Although some studies have shown that zinc ion intake might suppress tumor growth, overwhelming reports focus on the promoting role of zinc in tumor initiation and development, or even driving metas44tasis. MT2A, acting as a zinc donor, induces IGFBP2 and inhibits the expression of E-cadherin through a zinc finger protein.^454,455^ Recombinant IGFBP2 promoted migration and invasiveness of ESCC cells via NF-κB, Akt, and Erk signaling pathways.

**Fig. 8:**
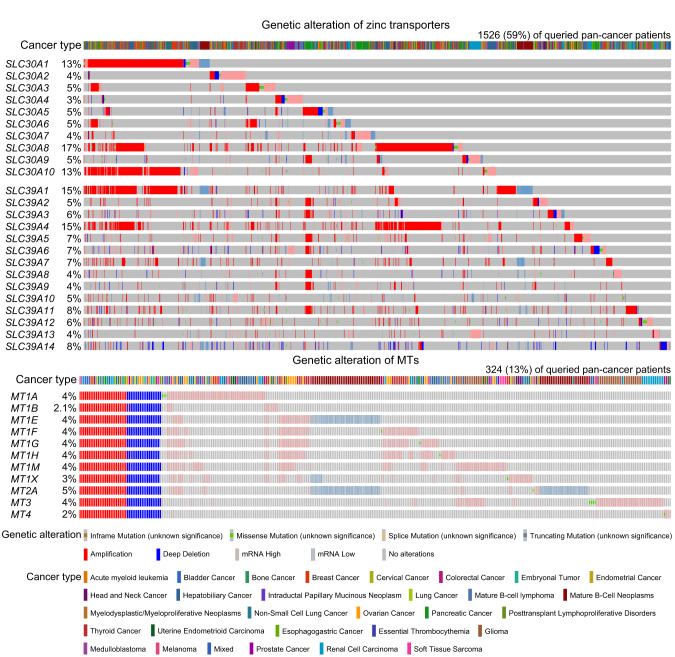
Genetic and mRNA alterations of zinc transporter and MT family genes in pan-cancer patients. The upper figure illustrates the gene alterations of zinc transporters. Out of the queried pan-cancer samples, 1526 (59%) showed copy number aberrations, mutations, and mRNA expression changes. The lower figure displays the gene alterations of MTs, where 324 (13%) of the queried pan-cancer samples demonstrated copy number aberrations, mutations, and mRNA expression changes. This diagram includes a total of 30 cancer types, marked with different colors. The data source is from the pan-cancer analysis of whole genomes dataset in cbioportal (https://www.cbioportal.org/study/summary?id=pancan_pcawg_2020)

In pan-cancer copy number variation (CNV) and mutation analyses from the TCGA database,^456^ most of the *SLC30* and *SLC39* family genes demonstrated gene amplification, especially *SLC30A8*, *SLC30A1*, *SLC30A10*, *SLC39A1*, and *SLC39A4*. Notably, the gene for *SLC30A8* and *SLC39A4* amplification was co-occurring in almost all cancer patients. Interestingly, the cases with *SLC39A14* deletion appear to be more than those with amplification (Fig. 8). Although ZIPs are more commonly regarded as oncogenes in cancer, prostate cancer is an exception. Studies also suggested that the function of the zinc transporters may be contradictory among different cancer types. As we delve into the gene alterations in MTs, our attention is captured by the astonishingly consistent variations observed among all MTs members (Fig. 8). Notably, the compelling set of data from representative tumor patients showcases the remarkably homogeneous trends in gene alterations among all MTs members. Such changes predominantly encompass amplifications and deep deletions, implying pivotal roles for MTs in the context of cancers. Despite the similar gene alteration trends, disparate mRNA expression profiles are observed for different MTs members. This intriguing observation suggests the involvement of intricate transcriptional regulatory mechanisms governing MTs genes. The diversity in mRNA expression levels might arise due to a myriad of factors, potentially linked to cellular context, tissue specificity, and even cancer types. Thus, research on zinc transporters and MTs in tumorigenesis is still a long way to go.

#### Other cancers

Zinc homeostasis disruption has been observed in patients with various types of cancers. Studies have highlighted the significance of zinc-containing enzymes called matrix metalloproteinases (MMPs), which can be activated by zinc.^457,458^ ZIP4, in particular, has been shown to regulate the expression of MMP2 and MMP9, influencing zinc concentration and promoting invasiveness and migration of hepatoma cells.^397^ Notably, ZIP4 expression is linked to post-liver transplantation outcomes in HCC patients, making it a potential treatment target and prognostic marker for liver transplantation in HCC cases.

Besides, ovarian cancer, the most lethal gynecologic malignancy, exhibits rapid progression and widespread metastases.^459^ Of note, ZIP13 was found to promote the proliferation, invasion, adhesion, and metastasis of ovarian cancer cells in vitro and in vivo.^73^ The underlying mechanisms involve intracellular zinc distribution disruption and activation of the Src/FAK pathway, ultimately leading to ovarian cancer metastasis.

Drosophila melanogaster serves as a powerful model for cancer biology studies. Drosophila ZnT7 (dZnT7) acts as a tumor suppressor, negatively regulating JNK signaling.^460^ dZnT7 knockdown induces JNK activation, promoting both cell-autonomous and nonautonomous autophagy, ultimately resulting in tumor overgrowth and migration.

Additionally, ZIP9 activation, through testosterone binding, induces an increase in cytosolic zinc in melanoma cells, thereby promoting cancer proliferation.^461^ In gliomas, MT3 plays a key role in autophagy flux regulation via zinc-dependent lysosomal acidification,^435^ contributing to glioma cell resistance to irradiation treatment. Targeting MT3 may thus enhance the efficacy of irradiation treatment. By elucidating the disruption of zinc homeostasis and its implications in cancer progression, these findings provide valuable insights into potential therapeutic strategies for diverse cancer types. Further research in this field may pave the way for improved cancer treatment and management.

### Cellular zinc metabolism in cardiovascular disease

Noncommunicable diseases, such as cardiovascular disease (CVD) and cancer, are the leading causes of death worldwide.^462^ The correlation between zinc and CVDs is a complex and multifaceted topic. Evidence suggests that zinc may be protective against certain CVDs, although the exact mechanisms are not fully understood.^270,463,464^ Here, we focus on elucidating the crucial involvement of zinc in the progression of CVDs, specifically with regard to atherosclerosis (AS), diabetic cardiomyopathy, myocardial ischemia/reperfusion (I/R) injury, and heart events.

#### Atherosclerosis (AS)

Hyperlipidemic environments and inflammatory factors are known to significantly contribute to the development of AS.^465^ Recent research highlights the critical role of ZD in the progression of this condition.^270^ Zinc exerts influence on various characteristic aspects of AS, including increased apoptosis and disrupted NO levels. NO, synthesized in endothelial cells (ECs), acts as an essential endothelium-derived vasodilator. Reduced availability of NO occurs when there is a decrease in the expression or activity of endothelial NO synthase (eNOS), actively participating in the atherogenic process.^466^ Additionally, it has been suggested that reduced NO generation in atheroprone regions, combined with increased ZnT1 and MT expression, may lead to decreased intracellular free zinc.^467^ Studies using Zip13-KO mice have shown elevated levels of the cardiac fibrosis marker Col1a1 and the vascular inflammation-related gene eNOS, indicating the physiological importance of ZIP13 in maintaining cardiovascular homeostasis by resolving inflammation and stress response.^468^

Moreover, the induction of EC apoptosis in response to oxidative stress is a characteristic atherogenic trait. Zinc is also associated with apoptosis and proliferation in vascular smooth muscle cells (VSMCs),^469,470^ the primary contributors to the composition of atherosclerotic plaques. The regulators ZnT3 and ZnT10 play crucial roles in VSMC senescence and are susceptible to downregulation by Ang II and zinc.^471^ Ang II signaling pathways become activated with age and contribute to developing AS and vascular senescence.^472^ Interestingly, decreased catalase expression is observed, leading to ROS accumulation and induction of senescence.^471^ ZnT3 and ZnT10 work to prevent increases in ROS levels by modulating the expression of catalase.

#### Myocardial ischemia/reperfusion (I/R) injury

Myocardial ischemia/reperfusion (I/R) injury is a prevalent cardiovascular condition associated with a high mortality rate.^473^ Recent studies have revealed the importance of zinc homeostasis in cardiomyocytes during reperfusion, as zinc loss upon reperfusion contributes to I/R injury.^474^ The crucial role of ZIP transporters in maintaining zinc homeostasis has been demonstrated, with ZIP2 playing a significant role in this process.^475^ Deletion of the ZIP2 gene notably intensified myocardial I/R injury, whereas upregulation of ZIP2 demonstrated the potential to mitigate I/R injury. These findings suggest that ZIP2 exerts a cardioprotective effect against I/R injury by restoring zinc homeostasis.^475^ Additionally, ZD has been shown to activate STAT3 through ER stress-induced Ca^2+^ release and subsequent CaMKII activation, enhancing the transcriptional activity of ZIP9 and protecting against cellular ZD.^476^

ZIP7 upregulation, on the other hand, hinders the accumulation of PINK1 and Parkin in mitochondria by increasing zinc outflow to the cytosol, contributing to the genesis of myocardial reperfusion injury by inhibiting mitophagy during reperfusion.^477^ Consequently, the upregulation of ZIP7 is considered a significant feature of myocardial reperfusion injury and may present a novel therapeutic target for myocardial reperfusion injury and other cardiac diseases caused by oxidative stress or mitochondrial dysfunction.

Endogenous ZnT-1 has been shown to have a substantial protective effect against I/R injury, which is mediated by the C-terminal domain of the protein through the activation of Ras-ERK signaling.^478^ Additionally, ZnT-1 serves various functions, such as binding to Raf1 and triggering the ERK cascade.^479,480^ Additionally, it hinders LTCC activity by interacting with the β-subunit of the voltage-dependent calcium channel. The significance of ERK cascade activation in promoting cell survival after I/R injury has been extensively recognized.^481^ Recent evidence suggests that nuclear factor (erythroid-derived 2)-like 2 (NRF2) activation/overexpression increases total zinc content in HCAEC with minimal changes in HCASMC, consistent with observed changes in ZnT1 and MT protein expression.^482^ This finding further highlights the complex interplay between zinc, ROS, and endogenous antioxidant defenses regulated by NRF2.

#### Diabetic cardiomyopathy (DCM)

DCM is a prevalent and severe complication of diabetes. A link between systemic ZD and the increased incidence of diabetes and diabetic cardiovascular complications has been established. Notably, in diabetic mice, zinc supplementation has been shown to significantly protect against the development of DCM through the induction of cardiac MT.^483–486^ MT has proven effective in countering cardiac fibrosis under stress conditions like diabetes and nicotine exposure.^484,487^

MTs offer cardiomyocytes protection primarily through zinc-dependent antioxidant effects. During the early stages of diabetes, cardiac mitochondria experience cytochrome c release-dependent apoptosis. However, MT substantially inhibits this early cardiac apoptosis caused by diabetes by suppressing mitochondrial oxidative stress, particularly the depletion of GSH, which significantly prevents the development of DCM.^483^ Moreover, MT suppresses Ang II-induced NOX-dependent nitrosative damage and cell death in both nondiabetic and diabetic hearts early in the injury process, effectively preventing the later development of Ang II-induced cardiomyopathy.^488^ Furthermore, MT ameliorates ROS generation and cardiac fibrosis despite persistent cardiomyocyte contractile and intracellular Ca2þ derangement.^489^ Both MT overexpression and direct MT administration can reduce DCM by suppressing peroxynitrite-derived nitrosative damage and ROS production in diabetic hearts.^490^

Recently, it has been demonstrated that zinc-induced cardiac endogenous antioxidant MT blocks TRB3 induction, thereby preserving Akt2 signaling and preventing DCM. The development of pharmaceutical inducers of cardiovascular MT holds promise as a preventive measure against cardiomyopathy in diabetic patients.^491^ In conclusion, the induction of MTs presents a potential therapeutic approach for preventing diabetic DCM.

#### Heart event

The zinc level in heart tissues is approximately 1 g or less, and it has been shown to have a positive correlation with ejection fraction in humans.^492^ At a concentration of 1 nM, zinc can directly activate RyR2, which has a much higher affinity for zinc than Ca^2+^ (about three-fold), providing an essential mechanistic explanation for the association between zinc dyshomeostasis and certain cardiomyopathies.^493,494^ ZnT-1 is an endogenous negative regulator of the LTCC, particularly in the heart, where it appears to participate in cardiac electrical remodeling following atrial fibrillation. Increased ZnT1 expression is observed in patients with atrial fibrillation.^495^ Mechanically, ZnT-1 was demonstrated to regulate the LTCC by interacting with its regulatory α1-subunit, thus limiting the plasma membrane expression of the LTCC.^151^

Furthermore, serum zinc levels could serve as a valid diagnostic indicator for acute myocardial infarction (MI).^496^ Meta-analysis data indicates that a lower dietary zinc intake is associated with an increased prevalence of coronary artery disease (CAD), and there is a direct relationship between zinc status and MI.^496^ ZIP13 is ultimately in charge of CaMKII mobilization, while the suppression of ZIP13 aggravates myocardial infarction through destabilizing mitochondrial signalings.^497^ Moreover, with respect to calcific aortic valve diseases, which is one of the most widespread heart valve disorders, the expression of ZIP13 is markedly enhanced. Correspondingly, ZIP13 knocking down resulted in the inhibition of human valve interstitial cells in an in vitro calcification model.^498^ Thus, alterations in ZIP13 expression may occur due to cardiac stress, which may induce CVDs or promote their pathogenesis. Additionally, it has been demonstrated that ZnT5 is associated with heart function, and its deficiency causes osteopenia and sudden cardiac death.^386^

During cardiac hypertrophy, the expression of ZIP2 was downregulated.^499^ Inhibiting ZIP2 leads to the induction of interferon regulatory factor (IRF) 7 expression, which, in turns, triggers the activation of ZIP2 development. As a result, IRF7 functions the role of a feedback regulator to modulate ZIP2 expression according to its activity. Based on serial transgenic mouse models, it has been confirmed that IRF7, IRF8, and IRF9 were anti-hypertrophy factors that are consistently down-regulated in cardiac hypertrophy and heart failure.^500–502^ To conclude, leveraging ZIP2 to modulate cellular zinc metabolism could offer an innovative approach for treating these two diseases.^499^

Besides, zinc emerges as a novel inhibitor of Calcific aortic valve disease (CAVD).^498^ The ZnR/GPR39 is reduced in calcified aortic valves from patients with CAVD. The anti-calcific effect of zinc on human valve interstitial cells (hVIC) calcification is, at least in part, mediated through the inhibition of apoptosis and osteogenic differentiation via the GPR39-dependent ERK1/2 signaling pathway. Additionally, ZIP13 and ZIP14 play important roles in hVIC in vitro calcification and osteogenic differentiation.^498^

Additionally, left ventricular noncompaction (LVNC) is a cardiomyopathy caused by arrested compaction, characterized by excessive trabeculation with deep intertrabecular recesses and thin compact myocardium.^503^ ZIP8 has been identified as a crucial factor in ventricular trabeculation and compaction, revealing a potentially novel regulator of ventricular myocardial development. As such, it may be included in the list of genes worth screening in patients with ventricular noncompaction or other diseases involving dysregulation of ECM degradation.^503^

In conclusion, the effects of zinc on cardiovascular disease are multifaceted. Understanding the mechanisms by which cellular zinc metabolism and regulatory mechanisms influence these processes has the potential to develop new strategies for the treatment of cardiovascular disease.

### Cellular zinc metabolism in autoimmune diseases

Zinc plays various roles in autoimmune diseases, including its function as an effector of the immune system, inflammation, and metabolism. As mentioned previously, the ZIP family, ZnT family, and MTs act as crucial regulators of zinc levels and are involved in developing different autoimmune diseases, such as the production of autoantibodies and inflammatory responses.

One specific autoimmune disease is type 1 diabetes, characterized by the destruction of pancreatic β cells mediated by T cells. Additionally, individuals with type 1 diabetes exhibit circulating autoantibodies targeting several β cell autoantigens.^504^ In 2007, researchers identified zinc transporter 8 autoantibodies (ZnT8A),^233^ which have since been recognized as one of the four major islet autoantibodies along with GAD65 autoantibodies (GADA),^505^ islet antigen-2 autoantibodies (IA-2A),^506^ and insulin autoantibodies (IAA).^507^ In prospective studies involving hereditary relatives at first-degree risk for individuals of type 1 diabetes, ZnT8A typically emerges around the age of 3-4 years and persists until the onset of clinical disease.^233,508^ ZnT8A serves as valuable markers for childhood-onset type 1 diabetes.^509^ It is noteworthy that ZnT8A usually develops later in young individuals compared to IAA and GADA. The presence of ZnT8A, as well as IA-2A and ZnT8A positivity, can identify individuals with prediabetes who are at a high risk of rapidly progressing to clinical type 1 diabetes.^510,511^ Moreover, the HLA class I A*24 allele, which is implicated in increased predisposition to type 1 diabetes, negatively correlates with the presence of ZnT8A at and before diagnosis, taking into account the age at onset.^512,513^

Studies have proved that CD8^+^ T cells in individuals with diabetes recognize a range of ZnT8 peptides in different regions of the protein, including the transmembrane/loop and C-terminal regions.^514,515^ Furthermore, isolated CD8^+^ T cells from individuals with diabetes show greater secretion of IFN-γ when stimulated by ZnT8.^516^ Most of the mature ZnT8A responses target the C-terminal region of the protein, while only 10% recognize the N-terminal region.^517^ Within the C-terminal region, ZnT8A can specifically target amino acid 325 of ZnT8, and this specificity is determined by the SLC30A8 polymorphism rs13266634.^518^ Interestingly, the higher frequency of ZnT8A in childhood-onset patients is primarily due to an increased number of patients with aa325-nonrestricted ZnT8A. Additionally, the amino acid encoded by the polymorphic codon 325 (Arg, Trp, Gln) plays a significant role in the humoral autoreactivity of this protein.^518,519^

In addition to T cells, a clinical trial discovered novel cryptic B cell epitopes in the ZnT8 autoantigen, which showed reduced levels of naturally occurring autoantibodies in diabetes.^520^ ZnT8A titers decreased rapidly following the initiation of diabetes, reflecting the continuous loss of β-cell mass.^511,521^ Although type 1 diabetes is commonly linked to other organic-specific autoimmune endocrine diseases, little evidence exists for a linkage between ZnT8A and markers of Addison’s disease (21OHA), autoimmune thyroiditis (TPOA), pernicious anemia (ATP4A-A), or celiac disease (TGA).^522^ These findings suggest that islet autoantibodies are not pathogenic in type 1 diabetes but rather a consequence of the immune-mediated destruction of β-cells. From a clinical perspective, reducing ZnT8 transport activity or down-regulating its cellular expression is proposed as an anti-diabetogenic strategy, mimicking the protective effect of SLC30A8 haploinsufficiency in humans.^523^

As previously mentioned, ZnT3 is crucial for transporting synaptic vesicular zinc, which can impact various signaling pathways downstream. Previous studies have suggested that zinc release/influx may be an initial event in the production of ROS induced by NADPH oxidase activation in experimental autoimmune encephalomyelitis (EAE). In mice, gene deletion of ZnT3 reduces the clinical symptoms of MOG35–55-induced EAE. This improvement is accompanied by reduced demyelination and the infiltration of encephalitogenic immune cells in the spinal cord. Furthermore, ZnT3 gene deletion inhibits the formation of EAE-associated aberrant synaptic zinc patches, MMP-9 activation, and disruption of the blood-brain barrier.^524^ Additionally, Penkowa and Hidalgo demonstrated MT2 could become a prospective treatment candidate in multiple sclerosis, since it reduced cytokine expression in the CNS and prevent apoptotic neuronal death in an EAE model.^525^

Genome-wide association studies have revealed an association between the SNP rs13107325 in SLC39A8/ZIP8 and Crohn’s disease.^197^ Furthermore, microarray data from rheumatoid arthritis (RA) patients have shown a significant increase in the expression of ZIP8 in peripheral monocytes compared to healthy controls.^526^ Monocytes and macrophages play crucial roles in the pathophysiology of RA by delivering enhanced costimulatory signaling and producing proinflammatory cytokines.^527^ Since ZIP8 is constitutively expressed in resting monocytes and macrophages, it suggests that ZIP8-mediated zinc influx promotes inflammatory conditions in RA. Therefore, ZIP8 may represent a potential therapeutic target for various inflammatory disorders.

In conclusion, the regulation of cellular zinc metabolism and the involvement of zinc transporters and MTs play crucial roles in autoimmune diseases. This provides valuable insights into potential therapeutic targets and strategies for managing these complex conditions.

### Cellular zinc metabolism in infectious diseases

Zinc, a divalent metal, holds a critical role in host-pathogen interactions by influencing microbial growth, pathogenicity, and the host’s immune defenses. Within innate and adaptive immune cells, two distinct and contrasting zinc-dependent mechanisms exist to combat pathogen invasion: nutritional immunity and zinc toxicity. Notably, nutritional immunity is a mechanism employed by immune cells to reduce the availability of zinc in the host, thereby hindering pathogen growth. In parallel, an excessive increase in zinc content within monocytes can induce zinc toxicity in pathogens, leading to their apoptosis. This intriguing interplay of zinc-related pathways highlights its multifaceted impact on the host-pathogen dynamic.

On one hand, nutritional immunity serves as a mechanism employed by immune cells to reduce the availability of zinc in the phagosome or cytoplasm, limiting its access and creating a phenomenon that restricts essential transition metal ions, including iron, zinc, selenium, and manganese, at the host-pathogen interface. This nutrient limitation strategy starves the invading pathogens.^292,528^ Notably, in vitro studies have demonstrated the potential of zinc limitation strategies to combat carbapenem resistance caused by zinc metallo-β-lactamases, as evidenced by the restoration of carbapenem susceptibility in *Acinetobacter baumannii* and improved survival in mice infected with *Aspergillus fumigatus* when pathogens were starved with zinc chelators.^529–532^ This approach may serve as an adjunctive therapy for difficult-to-treat pathogens like Aspergillus fumigatus. Laser ablation inductively coupled plasma mass spectrometry (LA-ICP-MS) has revealed that tissue abscesses caused by Staphylococcus aureus exhibit significantly lower levels of detectable zinc compared to the high zinc levels in surrounding healthy tissue.^533^ While the specific factors responsible for sequestering zinc within abscesses remain unknown, the absence of nutrient zinc within the abscess appears to represent an immune strategy to control infection. Interestingly, in response to zinc sequestration, bacteria have developed mechanisms to overcome this limitation by expressing high-affinity zinc transporters. These zinc uptake systems can be categorized into two groups. The first category includes zinc transporter families with homology to the highly affinity ZnuABC transport system of *Escherichia coli*.^534^ Additionally, both *N. gonorrhoeae* and *N. meningitides* express a specific zinc-import system called ZnuC, ZnuB, and ZnuA to improve intracellular zinc status.^535^ The second category of zinc transporters is analogous with the eukaryotic ZIP family transporters, but ZIP homologs are exclusively discovered in *Escherichia coli*.^534^

On the other hand, in certain infections like *Mycobacterium tuberculosis*, the zinc content in the phagosome is excessively increased, leading to zinc intoxication of the pathogen.^536^ When monocytes are stimulated with *Mycobacterium bovis* BCG cell wall, they induce ZIP8 expression, suggesting that extracellular zinc can be drawn in to fuel the host’s zinc poisoning strategy.^131^ Nutritional immunity and metal intoxication are feasible immune strategies to limit pathogen growth and control infection. Nutritional immunity primarily affects enzymatic and metabolic functions, while metal overload contributes to the generation of ROS, reactive nitrogen species, protein mismetallization, and subsequent respiratory arrest.^533,537–539^

In particularly, within macrophages, two lines of host defense are observed: zinc sequestration and zinc intoxication. Sequestration of zinc by MTs deprives pathogens of this essential nutrient, making them susceptible to killing by superoxide.^540^ Infection of macrophages with *M. tuberculosis* triggers zinc intoxication in both the host and the intracellular bacteria, indicating that the host-pathogen interaction disrupts zinc homeostasis in both organisms. The cytokines TNFα and IFNγ promote the accumulation of zinc in the phagosome of Mycobacterium avium-infected mouse macrophages, and phagosomal zinc levels increase over time in response to infection with Mycobacterium tuberculosis.^541^ *M. tuberculosis* infection also up-regulates ZnT1 expression in human macrophages,^542^ which probably facilitates the increase of zinc levels in macrophage phagosomes in conjunction with *M. tuberculosis*.^543,544^ Additionally, ZIP8 has been identified as a feedback controller of macrophage inflammatory responses.^196^ Its expression is upregulated by LPS and TNF, and the mechanism involves direct regulation by the transcription factor NF-κB. LPS also up-regulates ZIP14 mRNA from primary human macrophages, which acts as a limiting inflammatory response.^545^ Furthermore, *M. tuberculosis* possesses a counter-defense strategy that involves extruding incoming zinc via the P1B-type ATPase efflux pump, CtpC, to resist zinc toxicity.^542^ Mutant bacilli lacking CtpC are highly sensitive to zinc, rapidly accumulate the metal, and are killed by human macrophages. Macrophages adopt a similar zinc intoxication mechanism to challenge non-pathogenic *Escherichia coli*, indicating that zinc poisoning is a general defense strategy against intracellular bacteria.^546^ Mycobacterial infection causes a “burst of free zinc” within macrophages and increases the levels of zinc-binding proteins, MT1 and MT2, and ZnT1.^547^ Although macrophages are not yet proven to be capable of metallotoxicity against pathogenic *Neisseria* species, it has been shown that these immune cells can enhance zinc accumulation in cytoplasm and phagocytic vesicles through ZIPs.^131,140,548^ This suggests that host-induced zinc toxicity may be relevant to pathogenic *Neisseria* infection. Therefore, high levels of zinc within macrophages can directly exert bactericidal effects.

Above all, cellular zinc metabolism influences host-pathogen interactions through nutritional immunity and zinc toxicity, affecting pathogen growth and host defense mechanisms. Zinc modulation offers potential therapeutic targets in infectious diseases.

### Cellular zinc metabolism in neurodegenerative diseases

Zinc homeostasis alterations have been suggested to be closely associated with the development of certain neurodegenerative diseases.^66,549,550^ In patients with PD, AD, and amyotrophic lateral sclerosis (ALS), there is a significant increase in the zinc content within the cerebrospinal fluid. ZD, on the other hand, was demonstrated to impact neurogenesis as well as augment neuronal apoptosis, resulting in impaired learning and memory, highlighting the importance of elucidating the involvement of cellular zinc metabolism in the pathogenesis of these diseases.

Altered neuronal zinc handling plays a pivotal role in AD pathogenesis. Zinc released during neurotransmission was found to bind to amyloid-β peptides, accelerating the assembly of amyloid-β into oligomers that impair synaptic function.^551^ Multiple studies indicate that ZnT3 is crucial for reducing the risk of AD by facilitating the excretion of neuronal zinc.^40,552–555^ The expression level of ZnT3 in the cortex has been observed to decline with age in individuals with AD and in healthy individuals.^40,556^ Additionally, a rare copy number variant of the ZnT3 gene may be involved in the monogenic determination of autosomal dominant early-onset AD. Metal chaperones such as CQ and PBT2, which maintain metal ion homeostasis, have been shown to restore cognition, elevate zinc levels in the hippocampus, and restore levels of key proteins involved in learning/memory and synaptic plasticity in ZnT3 knockout mice.^552^ This raises the interesting question of whether metal chaperones could serve as an alternative zinc transporter. It has been found that other transporters, such as vGlut1, may compensate for the deficiency of ZnT3 by loading zinc into synaptic vesicles.^557^ In turn, Lang et al.^558^ demonstrated that overexpression of the Drosophila homolog of human ZIP1 leads to zinc accumulation in Aβ42-expressing fly brains, and inhibition of ZIP1 expression reduces Aβ42 fibril deposits and improves cognition 16. Zinc binding to amyloid-β is also influenced by MT3 released by astrocytes. Furthermore, the decreased extracellular levels of MT3 observed in AD may facilitate hypermetallation of amyloid-β by zinc.^559^ A study utilizing microarray data from the human frontal cortex has shown that the expression of ZNT3 and ZNT4 significantly decreases with age, while the expression of ZIP1, ZIP9, and ZIP13 significantly increases.^67^

In vitro observations have confirmed the high enrichment of zinc within senile plaques. AD patients exhibit changes in ZnT proteins (ZnT-1, ZnT-4, and ZnT-6) 5. ZnT1 and ZnT4 are expressed throughout the senile plaque, whereas ZnT3, ZnT5, and ZnT6 are localized to the periphery of the plaque.^560^ ZnT10 mRNA expression is significantly decreased in the frontal cortex of patients with AD,^561^ similar to the case in APP/PS1 mice. Dysfunction of ZnT10 may contribute to Aβ deposition and the formation of senile plaques. Recently, research has shown that ZIP9 plays a key role in the effects of DHT in APP/PS1 mice.^562^ Specifically, ZIP9 influences the expression levels of synaptic proteins, including PSD95, drebrin, and SYP. It also affects dendritic spine density in the hippocampus. These changes are mediated through the ERK1/2-eIF4E signaling pathway, which in turn has an impact on learning and memory processes. Therefore, new experimental evidence suggests that androgen supplementation improves learning and memory in AD.

In addition to AD, alterations in intracellular zinc homeostasis are considered a critical factor in the development of PD. Overwhelming evidence supports the notion that excessive intracellular zinc levels are implicated in the development of the disease.^34,563^ Zinc directly interacts with α-synuclein, a causative agent of PD and other neurodegenerative diseases, promoting its aggregation.^564^ Furthermore, zinc released from corticostriatal terminals may predominantly contribute to the deleterious effects associated with motor and cognitive symptoms in PD, as it acts synergistically with glutamate.^207^ Excessive glutamatergic corticostriatal transmission has long been recognized for its contribution to the development of PD symptoms and neurotoxicity, leading to neuronal degeneration.

The relationship between zinc levels and Huntington’s disease (HD) presents contradictory findings. Synaptic dysfunction significantly contributes to the pathogenesis of HD,^565^ with vesicular zinc playing a significant role in synaptic function.^566,567^ Specifically, increased levels of zinc have been measured in HD patients, suggesting that mutant Htt (mHtt) may disturb zinc metabolism.^568^ mHtt decreased ZnT3 expression by suppressing the conjugation of Sp1 with ZnT3 promoter.^569^ As a result, it downregulates vesicular zinc levels in the brains of N171-82Q HD transgenic mice. However, ZD was observed in the hippocampus and cortex of the R6/1 mouse model of HD.^570^ Previous studies have demonstrated significantly higher zinc levels in the cerebrospinal fluid of patients with ALS. Likewise, the protein levels of ZnT3 and ZnT6 are markedly and significantly reduced in the spinal cords of ALS patients, while ZnT5 levels show a tendency to decrease, although not significantly.^571^ Importantly, dysregulation of zinc has recently been identified to be a possible procedure causing the disequilibrium in the nucleocytoplasmic distribution of SFPQ in neurodegenerative disorders, consisting of both AD and ALS.^572^ SFPQ, an omnipresent nuclear RNA-binding protein intricately involved in diverse facets of RNA genesis, has been closely associated with neuropathological disorders, including AD and ALS.^573,574^

In conclusion, cellular zinc metabolism appears to play a crucial role in the pathogenesis of neurodegenerative diseases. Altered zinc homeostasis can lead to the formation of senile plaques in AD and contribute to α-synuclein aggregation in PD. ZD and dysregulation have been implicated in synaptic dysfunction and impaired learning and memory. Understanding the intricate relationship between zinc and neurodegenerative diseases may offer potential therapeutic strategies for managing these conditions.

## Therapeutic targets for cellular zinc metabolism

In the realm of medical research, identifying and understanding therapeutic targets for cellular zinc metabolism has become an intriguing area of study. The delicate balance of zinc within cells is critical for maintaining various cellular processes and overall physiological well-being. In this essay, we delve into the significance of therapeutic targets related to cellular zinc metabolism, shedding light on their potential implications for human health and developing novel therapeutic interventions.

### Zinc transporters

#### Therapeutic potential of zinc transporters in carcinogenesis

Zinc transporters not only contribute significantly to the onset and progression of cancer, but they are also implicated in the development of both chemoresistance and radiotherapy resistance. This positions zinc transporters as potential targets for breakthroughs in cancer therapy. Current therapeutic strategies primarily focus on the ZIP family of transporters, employing a variety of approaches, including antibody-drug conjugates (ADCs), siRNAs, and natural inhibitors (Table 1). These therapies have demonstrated promising efficacy, and as a result, we posit that the targeting of zinc transporters may emerge as a focal point in the development of future anticancer drugs.

**Table 1:** The expression levels, clinicopathological correlation, and potential small molecules for zinc transporters in carcinogenesis

Member	Cancer type	Expression	Diagnostic marker	Prognostic marker	Small molecules	References
ZIP4	HCC	Upregulated	–	√	–	^397^
	Gliomas	Upregulated	√	√	–	^679^
	HGSOC	Upregulated	√	–	–	^585^
	PC	Upregulated	√	√	–	^391,393,394,396,399^
	NPC	Upregulated	√	√	–	^77^
	NSCLC	Upregulated	–	√	–	^583^
ZIP5	ESCC	Upregulated	–	–	miR-193b	^596^
ZIP6	ESCC	Upregulated	–	√	–	^447^
	BC	Upregulated	√	√	SGN‐LIV1A/LV (NCT01969643, NCT03310957, NCT03424005, NCT01042379, NCT04032704, NCT02093858)	^156,601,680^
					ZIP6‐Y antibody	^156^
					Faslodex, 4‐hydroxytamoxifen	^157^
					M1S9	^602^
ZIP7	BC	Upregulated	√	√	DMAT, TBB	^168^
	T-ALL	Upregulated	–	√	NVS‐ZP7‐4	^603^
ZIP9	HCC	Upregulated	–	√	–	^681^
	Bladder cancer	Upregulated	–	√	Dutasteride	^604,605^
	Melanoma	Upregulated			Bicalutamide	^461^
ZIP10	Osteosarcoma	Upregulated	–	√	666-15, GSK690693	^589^
	BC	Upregulated	–	√	ZIP10B antibody	^156,334^
	GC	Upregulated	–	√	XYA-2	^682^
ZIP13	Ovarian cancer	Upregulated	–	√	–	^73^
ZIP14	CRC	Upregulated	–	√	–	^417,683^

The development of chemoresistance often limits the success of anti-cancer treatments. The acquired resistance is driven to some extent by intra-tumor heterogeneity, mainly directed by cancer stem cells (CSCs).^575^ Moreover, the difference between CSCs and non-CSCs within the tumor microenvironment may be primarily attributable to a cell biological procedure called EMT.^576,577^ Activation of the EMT program enables tumor cells to resist the therapeutic agents, which is consistent with the attribute of CSCs.^578,579^ As previously mentioned, zinc transporters are pivotal in cell stemness and EMT programs, reflecting their function in chemoresistance. For example, ZIP4 increases gemcitabine resistance primarily due to the activation of ZEB1, via p-STAT3 in PC cells.^395^ In other words, ZIP4 upregulated the expression of ZEB1 in PC, which in turn induced a substantial downregulation of gemcitabine uptake protein ENT1 by integrin α3β1, ultimately limiting drug internalization through activation of the MAP kinase JNK. Besides, ZEB1 has also been proven to confer PC drug resistance by suppressing miR-20331.^401^ Nabhan et al. found that gemcitabine activity requires caspase activation in multiple myeloma.^580^ Interestingly, ZIP4 regulates PC cell apoptosis through the cleavage of caspase.^581^ So far, gemcitabine-based therapies have remained the standard of practice for treating advanced PC.^582^ Obviously, ZIP4 knockdown combined with gemcitabine may be another promising novel approach for the treatment of PC metastasis and drug resistance.

Moreover, another study substantiated that ZIP4 facilitates EMT of NSCLC. The mechanism is through activation of the Snail-N-cadherin pathway.^583^ Similarly, siZIP4 evoked an epithelioid phenotype in NSCLC, reduced the expression of CSC markers, and elevated cisplatin sensitivity.^584^ In contrast, within high-grade serous ovarian cancer (HGSOC), the overexpression of ZIP4 increased chemoresistance to cisplatin and doxorubicin.^585^ Mechanistically, ZIP4 is an upstream regulator of NOTCH3, a storable signature of CSC in HGSOC. NOTCH3 can regulate proliferation, acid resistance, and drug resistance in carcinomas.^586,587^ Currently, developing more efficient siRNA delivery techniques is an active segment of ovarian cancer research,^588^ and targeting ZIP4 holds excellent promise. In a study on osteosarcoma, the authors found that ZIP10 expression is induced by chemotherapy and that subsequent increased intracellular zinc content activated CREB and promoted ITGA10 expression.^589^ Notably, ITGA10 predicted poor osteosarcoma survival because it could promote chemoresistance through PI3K/AKT signaling. Strikingly, the CREB inhibitor 666-15 as well as another small molecule, the PI3K/AKT inhibitor GSK690693, attenuated chemoresistance in the cancer cells with ZIP10 overexpression.

In addition to mediating chemoresistance in tumor cells, zinc transporters also contributed to chemoresistance mediated in stroma cells. It has been reported that interstitial space connection between cancer cells and matrix cells might underpin tumor proliferation and chemoresistance.^590,591^ In the tumor microenvironment of the lung cancer model, the ZIP1^+^ CAF subgroup is enrichment after chemotherapy and developed potent gapped junctions with tumor cells via up-regulation of the CX43 protein.^592^ This study described a fascinating zinc recycling procedure. Chemotherapy induces necrosis in dying cancer cells and releases unstable zinc to the extracellular compartment. In chemotherapy, tumor cells are inhibited from taking up zinc from the extracellular space, which may lead to ZD in tumor cells. However, ZIP1^+^ fibroblasts have the ability to serve as zinc reservoirs, allowing the transfer of zinc from fibroblasts into tumor cells, and leading to the induction of ABCB1-mediated drug efflux and chemoresistance. In summary, zinc transporters exert an imperative effect in the tumor microenvironment, helping cancer cells to generate chemoresistance by regulating zinc concentration.

It is well-documented that radiotherapy induces cancer cell apoptosis by DNA damaging. Zinc is essential for the protection of cells against DNA damage, and its role appears to be enhanced in cancer cells.^593,594^ ZD significantly influences cell cycle.^595^ For example, in ESCC, miR-193b modulates the expression of ZIP5 and Cyclin D1.^596^ In ZD, miR-193b was observed to be silenced by methylation, which increases ZIP5 expression. Subsequently, ZIP5 overexpression enhanced cellular zinc content, thereby diminishing the DNA damage from radiotherapy.^596^ Additionally, radiotherapy resistance is a major barrier limiting the favorable prognosis in NPC as it may lead to tumor recurrence.^597^ Zeng et al. found that raised ZIP4 expression activated the PI3K/AKT pathway to induce EMT in NPC cell line C666-1.^77^ Accordingly, ZIP4 inhibition augmented radiation-induced apoptosis of C666-1 cells ex vivo and in vivo. Crucially, targeting ZIP4 in conjunction with radiotherapy may be an effective new therapy for treating NPC.^77^

ADC is a novel anti-cancer drug consisting of a monoclonal antibody coupled with a cytotoxic drug via chemical linker.^598^ ZIP6 is the cell surfacing target that is critical in cancer progression, which is undoubtedly the best candidate for ADC therapy.^599,600^ As a result, inhibitors of ZIP6, a promising target, are being developed. For example, Seattle Genetics (SGN)-LIV1A or ladiratuzumab vedotin (LV), is currently in clinical trials for metastatic BC.^156,601^ LIV-1, also called ZIP6, is a transmembrane protein overexpressing in BC. As an ADC, (SGN)-LIV1A is composed of an antibody that specifically binds to ZIP6 on BC cells and a potent cytotoxic drug payload. Upon binding to ZIP6-positive BC cells, (SGN)-LIV1A delivers the cytotoxic drug directly to the cancer cells, inducing cell death.

In addition to ADCs targeting ZIP6, a few small molecules have been reported. For instance, M9S1 extracted from Moringa oleifera significantly downregulated the expression of ZIP6 in MDA-MB-231 tumor,^602^ treatment with STAT3 inhibitor peptide, cell-permeable (#573096, Sigma). Besides, the antiestrogens (Faslodex and 4‐hydroxytamoxifen, each 100 nM) also indicated that the expression of LIV-1 was decreased in MCF7 cells.^157^ Through phenotypic screening of compounds, a ZIP7 inhibitor, NVS-ZP7-4, was identified that dominates the Notch signaling pathway in T-cell acute lymphoblastic leukemia (T-ALL) cell lines and initiates apoptosis by inducing ER stress.^603^ Another research group found that the administration of CK2 inhibitors, such as DMAT (dimehtylamino-4,5,6,7-tetrabromo-1H-benzamidazole) or TBB (4,5,6,7-tetrabromobenzotriazole), inhibited the activity of ZIP7 and was well tolerated by cancer patients.^168^ Another advantage of targeting ZIP7 in cancer is that it inhibits the mobilization of a large amount of tyrosine kinases, preventing cancer cells from shifting into another signaling pathway for regeneration.^148^

Additionally, testosterone promotes melanoma proliferation through the activation of ZIP9.^461^ The classic FDA-approved androgen receptor inhibitor bicalutamide also inhibits ZIP9, thus the antagonist of the tumor-promoting role of testosterone in melanoma,^461^ suggesting that ZIP9 may be an effective target for melanoma and other cancers. Correspondingly, novel evidence shows that another androgen, dihydrotestosterone, can increase migration and invasion via ZIP9-mediated intracellular Gαi/MAPK/MMP9 signaling in bladder cancer.^604^ Furthermore, bladder cancer progression dependent on ZIP9 could be inhibited by dutasteride, a 5α-reductase inhibitor.^605^

Notably, the transcription factor STAT3 was strongly activated and related to a worse outcome in GC.^606^ XYA-2, a novel STAT3 naturally occurring product inhibitor, has recently been identified. It synergistically suppresses the expression of MYC and ZIP10 (two downstream genes of STAT3), which exerts an anti-carcinogenic activity.^431^ Furthermore, ZIP6/ZIP10 heteromer plays an essential role in zinc-induced mitosis, involving breast cancer proliferation.^156,334^ Therefore, targeting the ZIP6/ZIP10 heteromer could be a significant approach to inhibit breast cancer invasion. Nimmanon et al.^156^ utilized ZIP6 residues 240–253 (ZIP6-Y) and ZIP10 residues 46–59 (ZIP10B) to target ZIP6 and ZIP10, preventing their heteromer formation and thereby impeding the progression of mitosis.

Most tumor-targeted therapeutic studies on zinc transporters have primarily focused on ZIPs, while fewer investigations have been conducted on ZnTs. However, several tumor types, such as pancreatic cancer^607^ and GC,^430^ exhibit low expression levels of ZnTs. Targeting low-expressed genes is a viable strategy. Gene therapy techniques,^608^ such as viral vectors or nanoparticle-based delivery systems, could be employed to deliver ZnTs specifically to tumor cells, enhancing the expression of these low-expressed transporters and providing a targeted therapeutic effect. Alternatively, nanoparticle-based delivery^609^ of ZnT’s activators may offer a targeted therapeutic approach. By targeting low-expressed zinc transporter proteins, especially members of the ZnT family, a novel perspective emerges to dysregulate zinc homeostasis in cancer cells.

Thoughtfully, zinc plays an essential physiological function in cells, presenting a dual impact in tumor therapy. Targeting zinc or zinc transporters for tumor therapy shows promise, but potential toxic effects must be considered. Inhibiting zinc transporters or chelating zinc can disrupt vital processes for cancer cell survival and proliferation, displaying potential as an anticancer strategy. Obviously, the potential of targeting zinc transporters in cancer therapy has been identified, and the development of targeted small molecule drugs for clinical cancer patients is imminent. The small molecules potentially targeting the aberrantly activated zinc transporters have been summarized in Table 1. However, zinc’s significance in normal cellular functions, including DNA repair^610^ and immune responses,^611^ warrants caution to minimize off-target toxic effects. Precise optimization of zinc-targeted therapies is necessary to achieve tumor-selective cytotoxicity without harming healthy tissues. Understanding zinc’s specific molecular mechanisms in tumorigenesis is pivotal for developing low-toxicity targeted therapies.

#### Targeting zinc transporters in other diseases

While much of the current research on zinc transporter targeting has been concentrated on tumoral diseases, these essential proteins are not limited to oncological applications. Emerging evidence reveals their significant potential as therapeutic targets for a spectrum of disorders, including anemia, diabetes, malignant muscular dystrophy, and liver fibrosis. Table 2 summarizes the clinical value of targeting zinc transporters beyond cancer therapy.

**Table 2:** Possibility for targeting zinc metabolism in multiple diseases

Protein	Disease	Expression	Current or potential targeting value
ZIP5	Diabetes	Downregulated	The potential therapeutic target for diabetes-related diseases.^185^
ZIP10	Hematopoietic disease	Downregulated	Targeting ZIP10 may be a new therapeutic strategy against early fetal anemia.^50^
ZIP14	Dystrophic muscles	Upregulated	Underscores the importance of regulated zinc homeostasis in metastatic cancer-induced muscle dystrophy and suggests a novel treatment avenue by targeting ZIP14.^50^
	Liver cirrhosis	Upregulated	A new potential therapeutic avenue for preventing iron-death-induced liver fibrosis.^185^
ZnT8	Diabetes	Downregulated	To achieve more accurate early classification of diabetes and identification of which patients will rapidly require insulin treatment (NCT02287506). The usefulness of Intermittently Scanned Continuous Glucose Monitoring in the Diagnosis of Maturity-onset Diabetes of the Young (MODY) Patients (NCT05918484). The Mechanism of TCF7L2 and ZnT8 on Antipsychotic-induced Metabolic Syndrome (NCT02093858). Study whether ZIP8 rs13266634 polymorphism is associated with T2DM susceptibility and study the effect of zinc supplementation on glycemic control in patients with type 2 diabetes (NCT03112382, phase 4).
MT1/2	AD	–	Modulation of MT-I/II expression is a potential therapeutic target to treat the onset and progression of cognitive impairment.^618–620^
	Ocular neovascularization	–	MT1/2 is a potential novel therapeutic target for diseases involving ocular angiogenesis.^50^

Emerging research involving two distinctive models, including zip10 mutant zebrafish as well as the hematopoietic Zip10-deficient mice, has made significant strides in our understanding of hematopoiesis.^50^ Intriguingly, both models demonstrated more pronounced hematopoietic impairment than their counterparts lacking transferrin receptor 1, an established iron-gatekeeper. Research outcomes suggest a larger effect of zinc than iron in early hematopoietic stem cells (HSCs), underlining the significance of ZIP10 and zinc homeostasis in promoting proliferation and differentiation of fetal HSCs. Thus, a new vista opens for developing of therapeutic strategies against early fetal anemia by targeting ZIP10.

As mentioned in the previous section, zinc and its transporter proteins are implicated in insulin synthesis, secretion, and utilization. A particular study shed light on Zip5, which was found to be down-regulated in pancreatic β-cells of a diabetic mouse model.^612^ Intriguingly, the study revealed that zinc influx via Zip5 induced Glut2 expression through the activation of Sirt1-mediated Pgc-1α, proposing Zip5 as potential therapeutic target for diabetes-related diseases. Additionally, zinc transporters, specifically ZIP14, seem to be potential game-changers in the treatment of malignant muscular dystrophy. A conspicuous upregulation of ZIP14 was observed in dystrophic muscles from metastatic cancer. Further investigation revealed that ZIP14-mediated zinc accumulations in differentiating muscle cells cause deletion of myosin heavy chain.^613^ This finding underscores the importance of zinc homeostasis regulation in metastatic carcinoma-induced muscular dystrophy and suggests new avenues for treatment by targeting ZIP14.

In the context of liver health, zinc and its transport proteins carry immense importance, particularly in cases of liver fibrosis or cirrhosis. A model of iron metabolism disorders, the Trf-LKO mouse model, was subjected to hepatocyte-specific Trf knockout.^614^ The absence of hepatic Zip14 expression reduced hepatic iron build-up, thereby alleviating iron-death-mediated hepatic fibrosis triggered by a high-iron diet or CCl4 injection. Notably, Zip14 can transport iron ions in addition to zinc ions, providing another potential therapeutic avenue for preventing iron-death-induced liver fibrosis. Above all, the diverse roles of zinc transporters underscore their potential as therapeutic targets. The continued exploration of these transporter proteins will likely yield more significant insights and open the door to a broader range of therapeutic applications.

### Therapeutic potential of MTs

MTs, by virtue of their metal-binding capabilities, are central to many physiological and pathophysiological processes. They notably regulate zinc and copper homeostasis, shield against oxidative stress, and detoxify heavy metals.^615–617^ The exploratory frontier of MTs as potential therapeutic agents has been pushed substantially in recent times.

Neurodegenerative disorders such as AD and PD often exhibit aberrant metal homeostasis and pronounced oxidative stress, paving the way for the potential therapeutic application of MTs.^618–620^ Despite the seemingly promising outlook, some investigations have paradoxically led to contrary outcomes. For instance, the Tg2576 mouse model for AD, when subjected to an MT1/2-deficiency, demonstrated a partial rescuing of mortality and body weight changes that were induced by the human amyloid precursor protein.^621^ In addition, a reduction in amyloid plaque burden has been observed across both the cerebral cortex and hippocampus, although the overall effects on amyloid cascade, neuroinflammation, and behavior are complicated because of the deletion of MT1/2.^622^ In another study focusing on ocular neovascularization, a contributory factor to blindness, MT1/2 was found to play significant roles in retinal and choroidal neovascularization. The authors proposed the potential of MT1/2 as novel therapeutic targets for diseases involving ocular angiogenesis.^623^

Furthermore, MTs have demonstrated potential applicability in cancer therapy. Abnormal MT expressions have been detected in numerous cancer types, often exhibiting a correlation between the level of MTs in tumor tissue and disease prognosis. In the context of CRC, MTs are commonly viewed as oncogenes. There is experimental evidence indicating SPINK1’s role in promoting tumor survival in CRC via the suppression of MTs.^624^ However, contrary studies have emerged, showing DC-SIGNR’s ability to encourage cancer cell metastasis in CRC through the promotion of MTs.^625^ These opposing findings underscore the intricate interplay between MTs and cellular mechanisms during cancer progression.

In conclusion, despite the clearly apparent therapeutic potential of MTs, their role is convoluted and context-dependent. To grasp fully the biological functions of MTs and to harness them effectively for therapeutic strategies, we require a profound understanding which can only come from further dedicated research.

### Zinc-based therapeutics and measurement

Beyond targeting cellular zinc metabolism components, the development of zinc-based therapeutics itself is a burgeoning field. Utilizing zinc ions or zinc complexes as therapeutic agents holds potential in various medical applications, including wound healing, antimicrobial treatments, and zinc supplementation for zinc-deficiency-related conditions. The clinical applications of zinc supplements, zinc chelators have been summarized in Table 3. Meanwhile, Table 4 summarizes the measurements of cellular free zinc.

**Table 3:** The clinical applications of zinc supplements and chelators

Disease	Dosage and species of zinc	Effect/Comments	Trial registration number	References
*Clinical applications of zinc supplements*				
Prediabetes	30 mg zinc gluconate/day, 90 days.	Zinc supplementation significantly decreased BMI and improved FPG, 2hpp, HbA1C, insulin, IS, and IR.	–	^684^
Type-2 diabetes	30 mg zinc sulfate/day, 6 months.	Zinc supplementation improved FBG and HOMA concentration. Beta cell function, insulin sensitivity and insulin resistance showed significant improvement as well.	–	^685^
	40 mg zinc sulfate/day, 12 weeks.	Zinc supplementation was observed on inflammatory marker concentrations or fold change in zinc transporter and MT gene expression.	NCT01505803	^686^
	50 mg zinc gluconate/day, 8 weeks.	The total antioxidant capacity was significantly elevated (16%) following zinc intake by patients with T2DM. The clinical and glycemic indices.	IRCT2015083102	^687^
Diabetes with thalassemia	25 mg zinc sulfate/day, 3 months.	Zinc supplementation improves glucose homeostasis in thalassemia.	NCT01772680	^688^
AS	45 mg zinc gluconate/day, 6 months.	Zinc supplementation reduced plasma CRP and IL-6 levels in men and women. Zinc may have a protective effect on AS because of its anti-inflammatory and antioxidant functions.	–	^689^
COVID-19	25 mg of elemental zinc as capsule/day, 15 days.	Oral zinc can decrease 30-day death, ICU admission rate and can shorten symptom duration.	NCT05212480.	^690^
COVID-19	15 mg zinc in an active product/day, 30 days.	The administration of an active product (ABB C1®) based on a combination of β-glucan and probiotic S. cerevisiae yeasts enriched with selenium and zinc in association with influenza and COVID-19 mRNA vaccines appeared to be able to stimulate trained immunity.	NCT04798677	^691^
Behcet’s disease	30 mg zinc gluconate/day, 12 weeks.	Zinc gluconate supplementation can be considered as an adjuvant therapy in alleviating inflammation and genital ulcer among Behcet’s disease patients.	–	^692^
	30 mg zinc gluconate/day, 12 weeks.	Zinc supplementation significantly improved non-ocular Behcet’s disease score and TLR-2 expression.	NCT05098678	^693^
HIV-1	10 mg zinc sulfate/day, 6 months.	Zinc supplementation does not result in an increase in plasma HIV-1 viral load and could reduce morbidity caused by diarrhea.	–	^694^
Cholera	30 mg zinc acetate/day, until resolution of diarrhea or for up to seven days.	Zinc supplementation significantly reduced the duration of diarrhea and stool output in children with cholera.	NCT00226616	^695^
Malaria	10 mg zinc gluconate/day, median follow-up: 331 days	Neither zinc nor multi-nutrients influenced malaria rates	NCT00623857	^696^
Thalassemia major	25 mg zinc sulfate/day, 18 months.	Zinc supplementation resulted in greater gains in total-body bone mass in young patients with thalassemia major.	NCT00459732	^697^
Hemodialysis	78 mg zinc gluconate/day, 2 months.	Zinc supplementation ameliorates abnormally high plasma Al concentrations and oxidative stress and improves selenium status in long-term dialysis patients.	–	^698^
	34 mg hemodialysis/day, 12 months.	Zinc supplementation reduces the erythropoietin responsiveness index in patients undergoing hemodialysis and may be a novel therapeutic strategy for patients with renal anemia and low serum zinc levels.	–	^699^
Head and neck cancers	25 mg Pro-zinc (a powder extracted from bovine prostate then chelated to zinc)/day, 2 months.	Zinc supplementation used in conjunction with radiotherapy could postpone the development of severe mucositis and dermatitis in patients with cancers of the head and neck.	–	^700^
Colorectal cancer	308 mg zinc sulfate/day, 108 days.	Zinc supplementation during chemotherapy cycles increased SOD activity and maintained vitamin E concentrations, indicating production of stable free radicals, which may have a positive effect on cancer treatment.	NCT02106806	^701^
	70 mg zinc sulfate/day, 16 weeks	Zinc supplementation on markers of oxidative stress in post-operative colorectal cancer during chemotherapy cycles.	NCT02106806	–
	Zinc gluconate, unknown dosage, 8 weeks.	Zinc supplement in regorafenib treated metastatic CRC patient (ZnCORRECT).	NCT03898102	–
	70 mg zinc sulfate/day, 4 months.	Modulation of immune response by oral zinc supplementation in chemotherapy for CRC.	NCT01261962	–
ESCC and GC	22.5 mg zinc oxide/day, 15.25 years.	Zinc supplementation was associated with increased total and stroke mortality.	–	^702^
GI cancer	Zinc sulfate, unknown dosage	Effects on quality of life with zinc supplementation in patients with GI cancer.	NCT03819088	–
*Clinical application of zinc chelators*				
Epilepsy	2 weeks 1 mg/kg/day clioquinol, 6 weeks 4 mg/kg/day clioquinol, 8 weeks.	To examine the potential anti-seizure activity of clioquinol in a small cohort of adolescents with drug-resistant epilepsy	NCT05727943	–
Hematological malignancy	800 mg clioquinol/day, 28 days.	To evaluate the dose-limiting toxicity, maximum tolerated dose, and recommended phase II dose of clioquinol in patients with relapsed or refractory hematologic malignancies.	NCT00963495	–

**Table 4:** Measurement and tracking methods for subcellular zinc

Category	Name	Kd	Targeted organelles	References
FRET	Zif	1 μM (pH = 7.4)	–	^703^
	ZapCY1	2.5 pM (pH = 7.1)	Golgi, ER, mitochondria	^672,678^
	eCALWY-4	630 pM (pH = 7.1)	ER, mitochondria	^675^
	eZinCh-2	1 nM (pH = 7.1)	ER, mitochondria	^676^
	GZnP1	58 pM (pH = 7.4)	–	^704^
BRET	BLZinCh-1	160 ± 29 pM (pH = 7.1)	ER, mitochondria	^671^
	BLZinCh-2	117 ± 16 pM (pH = 7.1)	ER, mitochondria	^671^
	BLZinCh-3	15.6 ± 1.0 pM (pH = 7.1)	ER, mitochondria	^671^
LMW	FluoZin-3-AM	15 nM (pH = 7.1)	ER, mitochondria	^705^
	Zinpyr (ZP)	10.2 nM (pH = 7.5)	Golgi, mitochondria	^706^
	ZnAF	2.7 nM (pH = 7.1)	–	^707^
	RhodZin-353	–	Mitochondria	^708,709^
	ZIrF	11 nM (pH = 7.0)	–	^710^
	TSQ	–	Cytoplasm	^711^

#### Zinc supplements

Zinc’s significance in maintaining overall health is extensively discussed in our review. Correspondingly, ZD results in developmental retardation of children, delayed genital development and hypogonadism, skin disorders, hair loss, teratogenic effects, as well as weakened immune function, leading to an increased susceptibility to infections.^626,627^ Given the wide range of essential biological functions zinc performs, addressing ZD through proper nutrition could make a huge contribution to various facets of human health.

The European Food Safety Authority has delineated different reference daily intakes of zinc for different population groups.^628–630^ Specifically, these intake guidelines prescribe a range of 9.4–16.3 mg for men, 7.5–12.7 mg for women, 9.1–14.3 mg for pregnant women, and a lower limit of 5.5–7.4 mg for children aged between 4 and 10 years. Furthermore, they propose an upper threshold for zinc intake, at 25 mg/day for adults, and 7–10 mg/day for children aged between 4 and 13 years, to prevent potential zinc toxicity. Regarding supplements or food fortification, the European Union has authorized several zinc compounds. Among these, zinc sulfate and zinc oxide stand out as popular choices due to their cost-effectiveness.^631,632^ Zinc sulfate, being water-soluble and comprising 23% zinc, and zinc oxide, though water-insoluble but containing a substantial 80% zinc, are extensively used.^632^ Concurrently, zinc citrate has emerged as a promising alternative due to its sensory attributes. This compound contains up to 31% zinc, is minimally insoluble in water, has no odor, and is relatively cost-effective, making it an ideal choice for supplementation.^633^ However, data regarding the absorption efficacy of these compounds in humans remains somewhat limited. Research in rats have shown that supplementation with zinc gluconate or zinc citrate resulted in a significant increase in zinc concentrations in the prostate, while zinc sulfate had no effect.^634^ Thus, understanding zinc intake recommendations and the efficiency of different zinc compounds for supplementation is crucial to fully optimize the benefits of zinc for various demographic groups. As further research unfolds, it will be important to monitor these developments, to refine and update guidelines accordingly.

Diabetics lose zinc due to increased urinary excretion, leading to diabetic complications. Zinc was described as having insulin-mimetic effects, so zinc supplements may be appropriate for people with diabetes.^635^ The ameliorative benefit of zinc supplements in diabetics can be summarized as the potential hypoglycemic effect of zinc, beneficial modulation of concomitant metabolic aberrations and impaired anti-oxidant status, and attenuation of renal lesions.^636,637^ A meta-analysis showed that zinc supplements dramatically reduced glycemic indices, including two-hour postprandial glucose, fast blood sugar (FBS), and hemoglobin A1c, in all randomized controlled trials.^638^ Zinc also has a favorable effect on blood lipids.^639^ In addition, low-dose (<25 mg/day), and prolonged (≥12 weeks) intake of zinc from supplements with potential biofortification may be beneficial in reducing risk factors for T2D and cardiovascular disease.^640^

In addition, under physiological conditions, zinc binds preferentially to MT, further activating MT to exert its anti-oxidative stress function. Studies have shown that zinc supplementation alleviates MT and oxidative stress in renal tissues of streptozotocin-induced diabetic rats, thereby preventing the development of diabetic nephropathy.^641^ Another animal study has shown that zinc supplementation, in particular, reduces the probability of hyperglycemia-mediated renal injury, which also involves the process of oxidative stress.^642^ Similarly, an animal study involving streptozotocin-induced diabetic rats has shown that zinc supplementation may protect against diabetes-induced peripheral nerve damage by stimulating MT synthesis and decreasing oxidative stress.^643^

Beyond MTs, zinc supplementation also significantly affects the expression of zinc transporters in diabetic patients.^644^ Interestingly, the mRNA expression of ZnT8, a transporter closely tied to insulin secretion and hence diabetic conditions, displayed considerable variability. Notably, higher levels of HbA1c, an indicator of long-term glucose control, were found in those participants who exhibited ZnT8 expression compared to their counterparts with no detectable ZnT8 expression.^644^ Besides, a positive correlation between the mRNA of ZnT5 and ZIP3 was observed exclusively among participants receiving zinc supplementation. However, the same supplementation seemed to nullify the correlation between ZnT5 and ZIP10. In addition to basic supplementation, recent research has made strides in applying zinc-based therapies for diabetes management. For instance, novel zinc coordination compounds^645^ and zinc oxide nanoparticles^646^ have been explored for their potential to improve clinical outcomes in diabetes.

Diarrhea leads to significant zinc loss, and zinc supplements have proven effective in their treatment.^647^ However, the exact mechanism underlying zinc’s therapeutic effects and its role in preventing subsequent morbidity remains unclear. This may be because zinc is indispensable in maintaining normal immune function.^648^ The WHO recommends zinc supplementation alongside oral rehydration salts for diarrhea management. Despite its benefits, zinc supplementation may lead to some side effects. In studies, infants and children receiving zinc gluconate (10 mg or 20 mg of elemental zinc, respectively) experienced more days with vomiting compared to the control group.^649^ Besides, one systematic review reported a higher risk of vomiting with zinc gluconate compared to zinc sulfate or zinc acetate.^650^ It has been suggested that the unpleasant taste of zinc contributes to vomiting, but this is more probably because of zinc’s gastric irritant properties.^651^

In fact, higher concentrations of zinc have been found to disrupt the absorption of other essential trace elements, especially copper.^652^ Consequently, patients with copper overload, such as those with Wilson’s disease, may gain from treatment with 50 mg of zinc acetate three or more times a day, which remains highly effective for up to 10 years.^653^ However, it is crucial to be cautious about potential adverse effects. One concern is that zinc supplementation could result in copper deficiency, in turn causing severe anemia and neutropenia.^654^ Moreover, supplementation with 80 mg of zinc per day for a week resulted in the suppression of mixed lymphocyte cultures in the body, demonstrating that high levels of zinc can impede immune function.^655^ Thus, to ensure the safe and effective use of zinc supplementation, it is recommended to limit the daily dose to no more than 25 mg.^640^ Higher dosages, especially extreme dosages of more than 75 mg/day, may increase the risk of developing aggressive prostate cancer.^640,656^ These findings are in line with the tolerable upper intake levels (ULs) of zinc set in both the Americans (40 mg/day) and Europeans (25 mg/day).^657^

#### Zinc chelators

In laboratory settings, researchers utilize specific zinc chelators to investigate processes that rely on zinc. One of the most used selective and membrane-permeable chelators for zinc ions is N, N, N’, N’-tetrakis (2-pyridinylmethyl)-1,2-ethanediamine (TPEN). TPEN exhibits the highest affinity for zinc compared to other chelators (Ka = 1015.58 M^−1^).^44^ Numerous reports have shown that depletion of zinc from cells through chelation is considered a potential cancer treatment strategy.^160,658,659^ However, it is essential to interpret zinc effects cautiously and assess their physiological relevance in such studies. TPEN’s strong zinc-binding affinity enables it to virtually eliminate the entire zinc response pool, a condition not attainable under normal or pathological circumstances, leading to predictable cell death.

In contrast, 2,3-dimercapto-1-propanesulfonic acid (DMPS), a heavy metal chelator, has the highest affinity for copper.^660^ Interestingly, DMPS has also been identified as a zinc chelator and has been found to effectively antagonize Zn^2+^-dependent snake venom metalloproteinases in vitro.^661^ Another widely used chelating agent is EDTA (Ethylenediaminetetraacetic acid), which forms stable complexes with various metal ions, including zinc.^662^ For example, in the context of therapeutic modulation in traumatic brain injury (TBI), zinc has emerged as a target.^663^ EDTA significantly increased the expression of neuroprotective genes and proteins after TBI.

Clioquinol, recently used as a topical agent for treating some skin infections, has drawn interest from researchers due to its zinc and copper chelating properties, making it a potential candidate for AD.^664,665^ The chelating activity of zinc appears to play a direct role in heme production.^666^ Both zinc and copper contribute to the deposition and stabilization of amyloid plaques, and chelators were shown to solubilize amyloid deposits.^667^ Notably, as zinc is essential for heme synthesis, which is recognized as increased in the brain of AD sufferers leading to oxidative stress, clioquinol’s binding to zinc reduces heme synthesis and oxidative stress.

#### Zinc measurement

The complexity of distinguishing protein-bound zinc from unbound zinc in experimental setups has led to the development and employment of various methods for specific investigations. The techniques used can be broadly divided into two categories: analytical methods and fluorescence techniques.

Analytical methods such as atomic absorption/emission spectroscopy and inductively coupled plasma mass spectrometry offer a relatively straightforward means of measuring total zinc, including both bound and unbound forms.^668^ These methods are particularly useful in obtaining a holistic view of zinc content within a given sample.

Moreover, fluorescence microscopy/spectroscopy is primarily employed to study the zinc pool without binding to protein. Two main fluorescence techniques are key in this aspect: low molecular weight (LMW) fluorescent/fluorogenic chelating agents (probes) and genetically encoded fluorescent proteins.^12^ Typically bifunctional and comprising both chelating agent and fluorophore, LMW probes function mainly on the principle of photo-induced electron transfer (PET).^669^ PET occurs among fluorophore and the chelating component, leading to fluorescence quenching, and this process is disrupted by zinc binding, leading to enhanced emission.^670^

Further advancements in fluorescence techniques have led to the common utilization of Förster Resonance Energy Transfer (FRET) and Bioluminescence Resonance Energy Transfer (BRET) sensors, both genetically encoded specifically for zinc.^671^ FRET sensors, with their inherently ratiometric nature, utilize interconnected donor as well as acceptor molecules, linked by a peptide sequence containing a zinc-binding domain.^669^ Changes in zinc concentration lead to conformational changes that alter energy transmission and affect the strength of the emission fluorescence.^669,672^ BRET, conversely, focuses on the transmission of energy across the fluorescent structural domains of the donor luciferase and the acceptor. Major advantages offered by BRET sensors are their resistance to photobleaching, absence of phototoxicity, and lack of background autofluorescence during measurement.^671,673^ These characteristics make BRET an invaluable tool for examining dynamic interactions and enzymatic activity in living cells.

Besides, specific genetically encoded sensors like CALWY, Zap/ZifCY, and those based on carbonic anhydrase are increasingly being used to gain enhanced control over intracellular zinc concentration and location.^674–678^ These sensors provide tailored advantages in managing intracellular variables, including concentration, localization, and calibration. Recently, a set of innovative organelle-targetable zinc fluorescent probes has been developed, comprising ZnDA-1H, ZnDA2H, and ZnDA-3H.^27^ These cutting-edge probes feature HaloTag ligand (HTL) molecules, which facilitate precise localization within specific organelles, and provide an excellent means of studying the physiological functions of the ZIP members residing in the ER and Golgi apparatus.

In conclusion, from comprehensive analytical methods to fine-tuned fluorescence techniques like FRET and BRET, researchers are now equipped with diverse tools that provide multidimensional perspectives on zinc’s behavior and interactions. The synthesis of these tools within a clinical context could revolutionize patient care, fostering a new era of precision medicine where zinc measurement and manipulation become critical components in disease prevention, diagnosis, and treatment.

## Conclusion and future direction

Undoubtedly, cellular zinc metabolism and zinc signaling are critical in a variety of biological functions, spanning from essential cellular processes to the development and progression of various diseases. Zinc acts as an essential modulator of cell homeostasis as well as is engaged in key signaling pathways that impact cell growth, proliferation, immune responses, and DNA repair. Dysregulation of zinc metabolism and signaling has been linked to numerous diseases, including cancer, neurodegenerative disorders, and infectious diseases.

Evidence suggests that a safe range of zinc intake is negatively associated with cancer risk. However, cancer cells inevitably require more zinc to maintain the oncogenic properties and metastasis, which functionally relies on the zinc transporter. Previous studies reported that the zinc transporter is aberrantly elevated and activated among multiple tumor types, particularly GI cancers. The significant upregulation of zinc transporters in GI cancers might be because that zinc absorption depends on the epithelial cells of the GI tract, which is the most vulnerable region for zinc homeostasis disorders. In BC and ESCC, zinc transporter ZIP6 is regarded as a diagnostic and prognostic biomarker. Similarly, ZIP10 is regarded as a cancer marker based on its methylation in CRC. Aberrant expression or hyperactivation of zinc transporters would also contribute to tumor resistance, which could be a malprognostic factor for cancer patients. Therefore, aiming at zinc transporters is expected to improve the efficacy of tumor therapies. Meanwhile, since zinc transporter proteins are predominantly distributed on cell membranes, developing small molecules or monoclonal antibodies for specific targeting is feasible.

Obviously, targeting zinc transporters offers potential strategies for treating various diseases, including cancer, neurodegenerative disorders, and infections. However, the study of zinc transporters is still at an infant stage. There are still several issues to be addressed, especially in cancer research. Firstly, the molecular mechanism for the expression of zinc transporters should be further elucidated. Nearly all the upstream regulatory mechanisms of the zinc transporter are still lacking. Thus, it is imperative to elucidate the critical transcriptional factors in regulating zinc transporter expression. Meanwhile, post-transcriptional and post-transcriptional regulation mechanisms need to be addressed. Next, several intellectual gaps still exist concerning the clinical relevance of zinc transporters and their downstream effectors in tumorigenesis. As the mechanisms of ZIPs and ZnTs are totally different in different cancer types, the detailed functional roles and underlying mechanisms are required to be comprehensively revealed. A comprehensive study of zinc transporter-related signaling might accelerate the development of combination therapeutic approaches specifically geared toward zinc transporters. Furthermore, apart from the cancer cell itself, the gut microbiota, including bacteria and viruses, has been implicated in playing a vital role in tumorigenesis and impacting the therapeutic efficacies of cancer patients, especially GI patients. We speculated that the gut microbiome might manipulate the zinc transporter expression and is involved in zinc-related signaling transduction. It will be a research focus on how the microbiome changes reshape the zinc transporters in tumor initiation and development. Finally, targeting zinc transporter is promising for eliminating cancer by developing small-molecule drugs and monoclonal antibodies. Notably, taking advantage of the fact that most zinc transporters are found to be localized on the membrane surface of cancer cells, targeting cancer cells with ADCs is also a potential therapeutic strategy. Meanwhile, it is required to carefully appraise the benefits and side effects of drugs targeting zinc transporters and develop novel delivery strategies. In conclusion, zinc transporters play multifaceted roles in solid tumors, and serve as diagnostic/prognostic tools and therapeutic targets.

Undeniably, the understanding of cellular zinc metabolism and zinc signaling is still evolving, and future investigations in this field are promising. The potential of zinc-based therapies, such as zinc supplements and zinc chelators, warrants exploration in the context of specific diseases. Understanding the optimal dosage, timing, and potential side effects of zinc supplementation or chelation will be crucial for the successful translation of these approaches into clinical practice. Besides, the detection of zinc levels and zinc-related molecular alterations in biological samples may serve as diagnostic biomarkers for various diseases, aiding early detection and guiding treatment decisions. In conclusion, research efforts in cellular zinc metabolism and zinc signaling will deepen the scope of our comprehension of fundamental biological processes and pioneer the way for emerging therapies to combat disease.
